# Robust phenotypic maintenance of limb cells during heterogeneous culture in a physiologically relevant polymeric-based constructed graft system

**DOI:** 10.1038/s41598-020-68658-z

**Published:** 2020-07-16

**Authors:** Mohammed A. Barajaa, Lakshmi S. Nair, Cato T. Laurencin

**Affiliations:** 1 Connecticut Convergence Institute for Translation in Regenerative Engineering, University of Connecticut Health Center, Farmington, CT 06030 USA; 2 Department of Biomedical Engineering, University of Connecticut, Storrs, CT 06269 USA; 3 Albert and Wilda Van Dusen Distinguished Professor of Orthopedic Surgery, Raymond and Beverly Sackler Center for Biomedical, Biological, Physical and Engineering Sciences, University of Connecticut Health Center, Farmington, CT 06030 USA; 4 Department of Orthopedic Surgery, University of Connecticut Health Center, Farmington, CT 06030 USA; 5 Department of Materials Science and Engineering, University of Connecticut, Storrs, CT 06269 USA; 6 Institute of Materials Science, University of Connecticut, Storrs, CT 06269 USA; 7 Department of Chemical and Biomolecular Engineering, University of Connecticut, Storrs, CT 06269 USA; 8 Department of Craniofacial Sciences, School of Dental Medicine, University of Connecticut Health Center, Farmington, CT 06030 USA

**Keywords:** Biological techniques, Biotechnology

## Abstract

A major challenge during the simultaneous regeneration of multiple tissues is the ability to maintain the phenotypic characteristics of distinct cell populations on one construct, especially in the presence of different exogenous soluble cues such as growth factors. Therefore, in this study, we questioned whether phenotypic maintenance over a distinct population of cells can be achieved by providing biomimetic structural cues relevant to each cell phenotype into the construct’s design and controlling the presentation of growth factors in a region-specific manner. To address this question, we developed a polymeric-based constructed graft system (CGS) as a physiologically relevant model that consists of three combined regions with distinct microstructures and growth factor types. Regions A and B of the CGS exhibited similar microstructures to the skin and soft tissues and contained rhPDGF-BB and rhIGF-I, while region C exhibited a similar microstructure to the bone tissue and contained rhBMP-2. Primary rat skin fibroblasts, soft tissue fibroblasts, and osteoblasts were then cultured on regions A, B, and C of the CGS, respectively and their phenotypic characteristics were evaluated in this heterogenous environment. In the absence of growth factors, we found that the structural cues presented in every region played a key role in maintaining the region-specific cell functions and heterogeneity during a heterogeneous culture. In the presence of growth factors, we found that spatially localizing the growth factors at their respective regions resulted in enhanced region-specific cell functions and maintained region-specific cell heterogeneity compared to supplementation, which resulted in a significant reduction of cell growth and loss of phenotype. Our data suggest that providing biomimetic structural cues relevant to each cell phenotype and controlling the presentation of growth factors play a crucial role in ensuring heterogeneity maintenance of distinct cell populations during a heterogeneous culture. The presented CGS herein provides a reliable platform for investigating different cells responses to heterogeneous culture in a physiologically relevant microenvironment. In addition, the model provides a unique platform for evaluating the feasibility and efficacy of different approaches for simultaneously delivering multiple growth factors or molecules from a single construct to achieve enhanced cell response while maintaining cellular heterogeneity during a heterogenous culture.

## Introduction

In recent years, the demand for finding alternatives to biological grafts in treating traumatic injuries that involve the loss of multiple tissues has been increasing due to the limited availability of autografts and the need for immunosuppression to allografts^1^. For this reason, there has been a notably growing interest in developing innovative approaches and strategies towards the regeneration of multiple tissue units simultaneously. This is evidenced by the increase in the number of publications in this context^2^. Despite the advances made, many challenges still remain.

The simultaneous regeneration of multiple tissues is a complex process and requires the presence of different cell phenotypes that are able to maintain their relative physiological functions and phenotypic characteristics within one complex and heterogeneous environment to enable the regeneration of the distinct multi-tissue units^3,4^. However, one of the current major challenges during the regeneration of multiple tissues is the ability to maintain the physiological functions and phenotypic characteristics of distinct types of cells in one milieu. When distinct types of cells are combined in one milieu, they rapidly interact mostly through paracrine signaling via endogenously secreted soluble factors such as cytokines and growth factors. These biochemical interactions are capable of altering cell behavior and causing specific responses to be induced without the need for a physical contact (i.e., apoptosis, phenotypic alteration, induction or inhabitation of cell division, etc.)^5^. For instance, Wang et al. showed that a monolayer co-culture of soft tissue fibroblasts and osteoblasts caused a reduction in fibroblasts growth and an increase in alkaline phosphate (ALP) activity, suggesting trans-differentiation of fibroblasts due to the paracrine effects mediated by osteoblasts during co-culture^6^. Similarly, it was found that the paracrine effects mediated by osteoblasts during co-culturing with skin fibroblasts in a monolayer system resulted in a reduction in fibroblasts growth and trans-differentiation into an osteogenic lineage^7,8^.

For this reason, research efforts have focused on strategies that can regulate cell behavior during a heterogeneous culture to achieve phenotypic maintenance over the different cell phenotypes to ultimately enable the simultaneous regeneration of multiple tissues. These efforts have demonstrated that culturing cells in three-dimensional (3D) milieu with biomimetic structural cues relevant to each cell phenotype can remarkably regulate their response and change the extent of how they interact through paracrine signaling. Many works have shown robust phenotypic characteristic maintenance over distinct cell phenotypes during co-culture when embedded in 3D constructs with cell-relevant microstructures compared to co-culturing them in monolayer based-systems. For instance, Sanchez et al. reported that chondrocytes showed a significant reduction in their relative gene and protein expressions when co-cultured with osteoblasts in a monolayer system^9^. In contrast, when the same cell phenotypes were co-cultured in a 3D construct with cell-relevant microstructures, the physiological functions and phenotypic characteristics of both cell phenotypes were well maintained^10^. This has also been evident in many further studies where providing biomimetic structural cues relevant to each cell population along the construct resulted in spatial control over the different cells’ phenotype, thereby, regulating their relative gene and protein expressions in a region-specific manner, and promoting the regeneration of their respective tissues simultaneously in vivo^11–14^. These observations have led to the notion that the dimension in which cells are cultured in is a crucial fate determinant during a heterogenous culture, and to the vague impression that culturing cells in monolayer drives abnormal cell function or trans-differentiation, whereas 3D culture elicits a more physiological state.

Besides the role of the 3D biomimetic structural cues, the mode in which growth factors are presented along the construct also plays a key role in maintaining the physiological functions and phenotypic characteristics relevant to each cell type during a heterogeneous culture^15^. Growth factors are soluble molecules each of which is capable of promoting their own set of biological effects, from specific to broadly overlapping activities^16^. However, stimulating a specific cell phenotype with improper singling can result in undesirable adverse events such as apoptosis, inhabitation of cell division, and phenotypic alteration or even trans-differentiation to an undesired lineage. For example, stimulating skin fibroblasts, soft tissue fibroblasts and muscle myoblasts with bone morphogenetic protein-2 (BMP-2) significantly reduces their growth and induces their trans-differentiation into an osteogenic lineage^17–20^. Hence, dominating the extent of how growth factors are presented throughout the construct may facilitate control over cell behavior during a heterogonous culture and prevent such undesirable adverse events.

Over the past decades, a wide range of approaches has been employed for the controlled delivery of growth factors from biomaterials^15^. Spatial and local presentation of growth factors has been shown to be an effective approach to electing region-specific control over cellular behavior during a heterogeneous culture^21^. This approach allows for presenting multiple factors, each at a specific region along the construct, for stimulating a targeted population of cells^21^. Many strategies have been pursued to allow for spatial and local presentation of growth factors in biomaterials. Strategies include chemical immobilization of growth factor into or onto the matrix, which involves the chemical binding or affinity interaction between the growth factor-containing biomaterial substrate and a cell, and physical incorporation that is achieved by the incorporation, diffusion and pre-programmed local release of growth factor from biomaterial into the surrounding tissue^21^. In all cases, these strategies spatially guided cellular response to the desired end in a region-specific manner^21^.

Based on the aforementioned observations and strategies, the current study seeks to investigate whether the physiological functions and phenotypic characteristics of distinct cell phenotypes derived from the limb such as skin fibroblast (SFs), soft tissue fibroblasts (STFs), and osteoblasts (OBs) could be maintained in a single construct exhibiting biomimetic structural cues relevant to each cell phenotype and controlled presentation of growth factors. The rationale for selecting these cell types is due to the inability of both fibroblast phenotypes to maintain their relative physiological functions and phenotypic characteristics when co-cultured with osteoblasts due to the robust paracrine effects mediated by osteoblasts in co-culture^6–8^. Therefore, we believe that enabling the phenotypic maintenance of these cell phenotypes on a single construct can be instrumental towards regenerating them simultaneously.

Regenerative Engineering is an interdisciplinary field that has been defined as the Convergence of Advanced Materials Sciences, Stem Cell Sciences, Physics, Developmental Biology, and Clinical Translation for the regeneration of complex tissues and organ systems^22–24^. At the forefront of this transdisciplinary approach is the study of epimorphic regeneration and understanding the morphogenetic cues involved in a regenerative limb to help develop translational strategies for tissue and organ regeneration^23,25^. The current work sought to address one of the main challenges associated with the simultaneous regeneration of multiple tissues—maintaining the cellular heterogeneity of multiple cell populations in a heterogenous cellular environment—by regenerative engineering a 3D polymeric-based graft system that contains spatial heterogeneity in structural cues inspired by the structural heterogeneity found in the native in vivo milieu.

**Figure 1:**
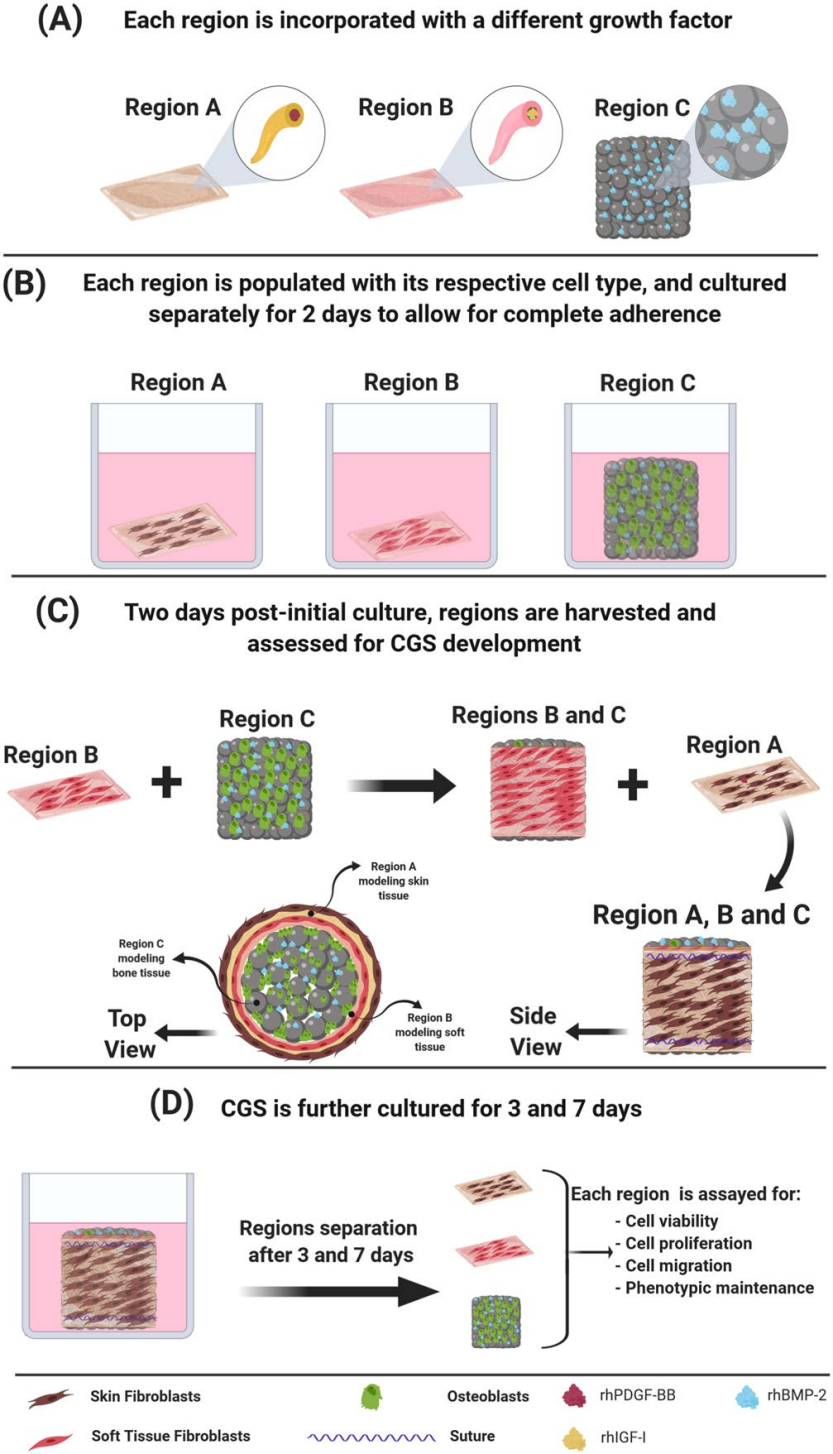
A schematic showing the various steps involved in the development of the CGS. (**A**) Regions A and B containing rhPDGF-BB and rh-IGF-I at the core–shell, respectively, and region C containing gelatin-mTG-rhBMP-2 hydrogel within the pore spaces. (**B**) Each region is populated with the relevant cell type and cultured separately in the optimal heterogeneous growth medium for 2 days to allow cells to completely adhere to their respective regions. (**C**) Two days post-initial culture, regions are harvested and assessed for CGS development in which region B is wrapped around region C and both regions B and C are wrapped by region A, followed by suturing the regions. (**D**) Post-CGS development, the CGS is further cultured for 3 and 7 days. At 3 and 7 days, CGS is harvested, regions are separated and assayed for cell viability, proliferation, migration, and phenotypic maintenance.

In this study, we developed a constructed graft system (CGS) that spatially modeled the 3D microenvironment of skin, soft tissue and bone. The CGS is composed of three combined regions, each exhibiting a different microstructure and contains a different growth factor. Region A of the CGS modeled the fibrous microstructure of the skin tissue and contained recombinant human platelet-derived growth factor (rhPDGF-BB), region B modeled the fibrous microstructure of soft tissues and contained recombinant human insulin-like growth factor-I (rhIGF-I), and region C modeled the interconnected and highly porous microstructure of the bone tissue and contained recombinant human bone morphogenetic protein-2 (rhBMP-2) (Fig. 1A). Both rhPDGF-BB and rhIGF-I were physically incorporated into their respective regions, while rhBMP-2 was covalently bonded to region C. SFs, STFs, and OBs were isolated from rats and seeded on their respective regions (Fig. 1B) followed by physically assembling the different regions into a 3D multi-layered configuration (Fig. 1C,D). Combining the different regions into this 3D configuration resulted in the production of a single construct with spatial heterogeneity in structure, growth factor type, and cell phenotype, mimicking the structural, cellular and chemical heterogeneity found in the native in vivo milieu. We first investigated the potential of biomimetic structural cues provided within the CGS to maintain the heterogeneity of the different cells in a region-specific manner during a heterogeneous culture. We next evaluated the feasibility of the CGS to act as a bioactive milieu by investigating its ability to contain and present different growth factors simultaneously to enhance the different cells’ physiological functions in a region-specific manner while maintaining distinct cellular regions. Our data suggest that the biomimetic structural cues provided within the CGS and the proper presentation of growth factors played a crucial role in ensuring heterogeneity maintenance of distinct cell populations during a heterogeneous culture. The presented CGS herein provides a reliable platform for investigating different cells responses to heterogeneous culture in a physiologically relevant microenvironment. In addition, the model provides a unique platform for evaluating the feasibility and efficacy of different approaches for simultaneously delivering multiple growth factors or molecules from a single construct to achieve enhanced cell response while maintaining cellular heterogeneity during a heterogenous culture.

## Results and discussion

### Regions with structural heterogeneity

The three different regions of the CGS were each fabricated using a different process to achieve regions with biomimetic structures relevant to each cell phenotype. Specifically, regions A and B were produced by electrospinning of poly (lactic-co-glycolic acid) (PLGA), and region C was produced by heat sintering of PLGA microspheres above its glass transition at 90 °C for 90 min^26^. PLGA was selected as the core material for the fabrication of the three different regions due to its biocompatibility and ability to be processed into different structures^27^. In addition, fabricating the three different regions using the same material was essential for our investigation to ensure that any changes in cellular behaviors observed during the study are due to the effects of the heterogeneous culture and/or the structural cues provided within the CGS and not due to variation in the type of material used.

**Figure 2:**
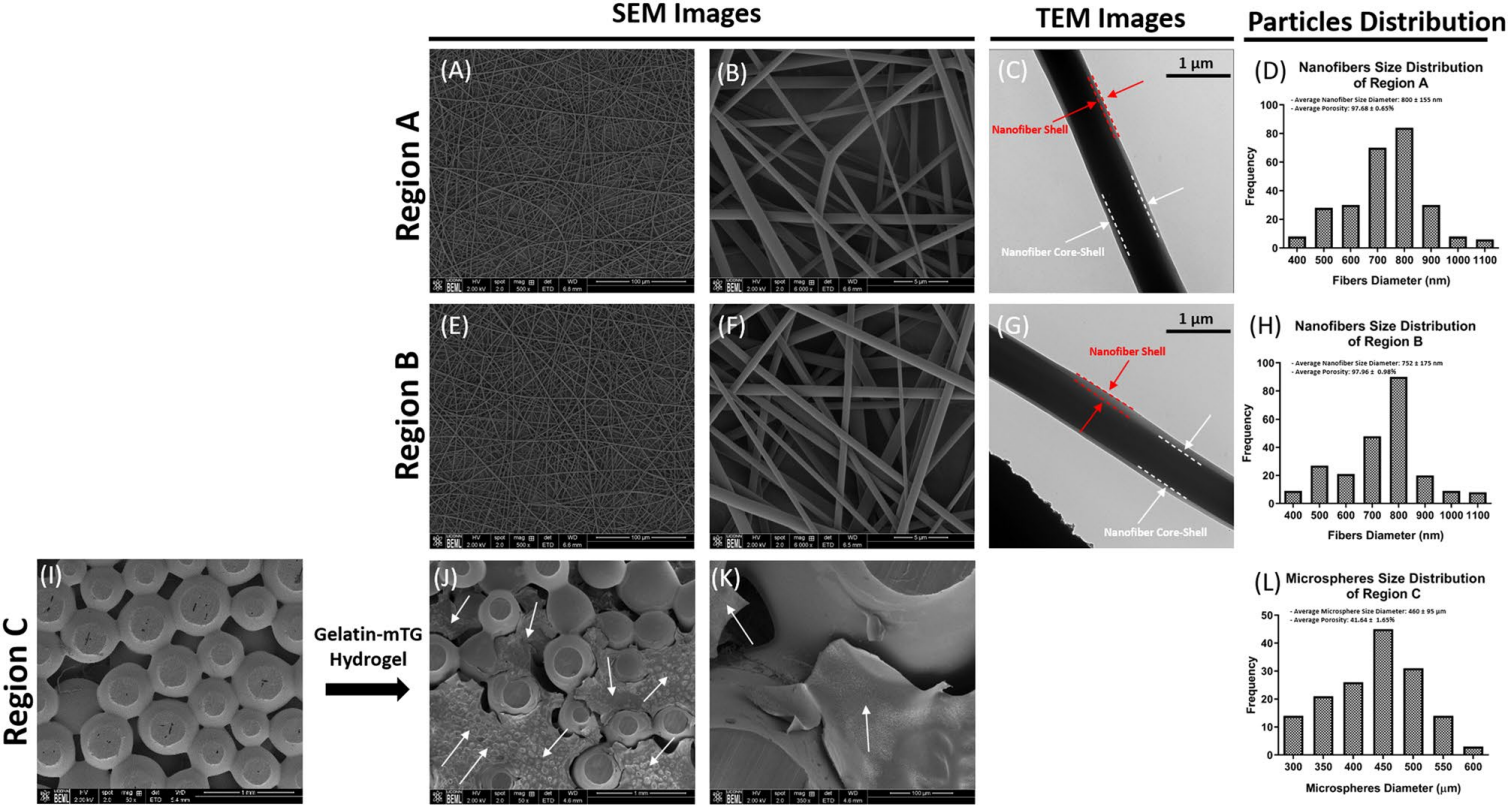
Morphology and properties of the different CGS regions. SEM images of regions A, B and C post-fabrication demonstrating the biomimetic structures to the corresponding native tissues as well as high porosities. (**A**,**E**) The overall morphology of the electrospun nanofibers for both regions A and B (scale bar 100 µm). (**B**,**F**) Higher magnification images showing the morphology of individual nanofibers and the pore spaces between them. Smooth nanofiber surface can be observed and a biomimetic fibrous structure similar to skin and soft tissues (scale bar 5 µm). (**C**,**G**) TEM images of PLGA-Gelatin Coaxial nanofibers with a clear core–shell structure, where gelatin is contained in the core and covered with a PLGA shell. (**D**,**H**) The size distribution of electrospun nanofibers in both regions A and B ranged between (400–1100 nm), mimicking the size range of collagen fibril found in the native skin and soft tissues. Both regions A and B exhibited high porosity with average pore volumes of 97.68 ± 0.65% and 97.96 ± 0.98%, respectively. The overall morphology of the sintered microspheres in region C (**I**) before and (**J**) after the incorporation of gelatin-mTG hydrogel into the pore spaces of the construct (scale bar 500 µm). (**K**) Higher magnification image showing the interconnectivity between microspheres and gelatin-mTG hydrogel occupying the pore spaces (scale bar 100 µm). (**L**) PLGA microspheres size distribution in region C ranged between (300 µm – 600 µm), resulting in an optimal pore volume for bone tissue regeneration (41.64 ± 1.65%). Red dotted lines represent the nanofiber shell (PLGA), white dotted lines represent the core–shell (gelatin) of the nanofiber, and white arrows represent gelatin-mTG hydrogel.

The gross appearance of all regions was examined using scanning electron microscopy (SEM) (Fig. 2). SEM images of both regions A and B show that the electrospinning process resulted in the production of a fibrous network that mimicked the fibrous microenvironment of skin and soft tissues (Fig. 2A,B,E,F). These synthetic nanofibrous structures have previously been shown to act as suitable platforms for the attachment and growth of SFs and STFs as well as the regeneration of their respective tissues^28–30^. Therefore, utilizing such structures in both regions A and B is feasible for addressing the current study’s questions.

Nanofibers in both regions A and B were obtained by coaxial electrospinning. This resulted in nanofibers with a core–shell structure as confirmed by the transmission electron microscopy (TEM) images (Fig. 2C,G), in which the core contains the growth factor solution and is covered by a PLGA shell. In this study coaxial electrospinning was selected over the conventional blend electrospinning (growth factors blended with the polymer solution during electrospinning) for the production of both regions A and B for its ability to (1) provide homogenous distribution of the incorporated growth factor throughout the fibers, (2) protect the bioactivity of the incorporated growth factors from the harmful effects of the electrical currents that are normally localized on the outer surface of the nanofiber during the electrospinning process, and most importantly (3) significantly minimize the initial burst release of the incorporated factor, as well as sustain, prolong, and localize its release within a limited domain^31–33^.

The SEM images of region C show that the heat sintering process of PLGA microspheres resulted in the production of an interconnected microsphere network that mimicked the microstructure and interconnectivity seen at the native bone tissue (Fig. 2I). The capacity of these sintered microsphere-based structures to support OBs growth, as well as bone regeneration has been well demonstrated in previous literature, making them an excellent choice for bone tissue regeneration applications^24,34–36^.

After the fabrication of region C, its pore volume was completely filled with gelatin-microbial transglutaminase (Gelatin-mTG) solution containing rhBMP-2, followed by incubation at 37 °C for 1 h to allow the gelatin-mTG-rhBMP-2 solution to completely gel within region C. Gelatin is naturally derived from collagen, is biocompatible, and favored by cells^37^. Gelatin-based hydrogels are generally used as carriers for the delivery of biomolecules such as rhBMP-2 due to their ability to control the release profiles of the incorporated factors depending on the crosslinking method^38,39^. Here, gelatin was used as the carrier for rhBMP-2 and was enzymatically crosslinked with mTG since this enzyme can initiate covalent bonding between gelatin and rhBMP-2^40^. Thus, the incorporation of the gelatin-mTG-rhBMP-2 hydrogel into the pore volume resulted in covalently bonded rhBMP-2 within region C. Figure 2J,K shows an even distribution of the incorporated gelatin-mTG-rhBMP-2 hydrogel within the pore spaces of region C (white arrows) indicating its successful incorporation.

To maintain the viability of the cells within CGS we modulated the porosity in all regions during the fabrication process to allow for sufficient oxygen and nutrient diffusion, as well as metabolic waste removal. This was done based on preliminary optimization studies performed in our laboratory that led to the selection of general electrospinning parameters^41^ as well as microsphere size distribution^42^ to yield constructs with high porosity throughout. As a result, all regions exhibited high porosity (97.68 ± 0.65%, 97.96 ± 0.98% and 41.64 ± 1.65%, respectively) (Fig. 2D,H,L).

### CGS development and in vitro evaluations of region-specific cell heterogeneity maintenance

Previous reports have shown that in 2D monolayer-based co-culture systems both SFs and STFs undergo trans-differentiation to an osteogenic lineage when co-cultured with OBs due to the robust paracrine effects mediated by OBs^6–8^. Therefore, in this study, we sought to investigate whether combining both fibroblastic phenotypes with OBs in a 3D physiologically relevant microenvironment can reveal different cell responses than what has been reported in the 2D monolayer-based cultures and can help regulate their relative physiological functions and phenotypic characteristics during paracrine interactions. We developed a CGS with biomimetic structural cues relevant to each cell phenotype. Developing the CGS was achieved by first seeding the three different cell phenotypes in their respective regions separately and culturing them for 2 days to allow for complete cell adherence. Prior to seeding the isolated primary cells, they were phenotypically characterized by immunofluorescent to confirm their lineages specificity (Supplementary Fig. S1). Two days post-initial seeding, the multiple regions were harvested and combined by assembling them into a 3D multi-layered configuration (Fig. 1). Combining the multiple regions into this 3D configuration resulted in the production of a single construct with spatial heterogeneity in structure and cell phenotype in a region-specific manner. The rationale for the graft system’s design, in terms of lacking the structural continuity between the different regions, is to allow the cells to only interact by paracrine factors while residing in their respective regions within the CGS. Thus, enabling the determination of the role of biomimetic structural cues presented within the CGS to maintain the different cells’ heterogeneity during paracrine interactions, and the potential of the CGS to act as a physiologically relevant milieu for studying different cell responses to heterogeneous culture.

Post-CGS development, it was further cultured for 3 and 7 days to allow for paracrine interactions to occur. During the 7 days, the CGS was cultured in a predetermined heterogeneous growth medium that was shown to support the growth of the three different cell phenotypes in a tri-culture (Supplementary Figs. S5,S6 and Supplementary Table S1). Predetermining the optimal heterogeneous growth medium for the three different cell phenotypes during the tri-culture was essential to ensure that any changes in cellular behavior was due to the effects of the paracrine interactions and/or the structural cues provided within the CGS and not due to the type of culture medium since the growth of cells could be affected depending on the type of medium used in culture^43^. Predetermining the optimal heterogeneous growth medium was achieved through the utility of a tri-culture system that was developed (Supplementary Fig. S2) in our laboratory and validated for its functionality (Supplementary Figs. S3,S4 and Supplementary videos S1,S2). Using this tri-culture system, we were able to determine an optimal growth medium that supported the growth of the three different cell phenotypes. While this tri-culture system was particularly developed and utilized in this study for determining an optimal growth medium that would support the growth of SFs, STFs, and OBs during heterogenous culture, it can also be used for the same purpose on other cell phenotypes, or for the purpose of studying the effects of the paracrine interactions between distinct population of cells in a monolayer environment for early-stage research investigations.

At 3 and 7 days, the ability of the CGS to maintain the region-specific heterogeneity during the heterogeneous culture was determined by harvesting the CGS, separating the regions and assaying each region for cell viability, proliferation, migration, and phenotypic maintenance.

**Figure 3:**
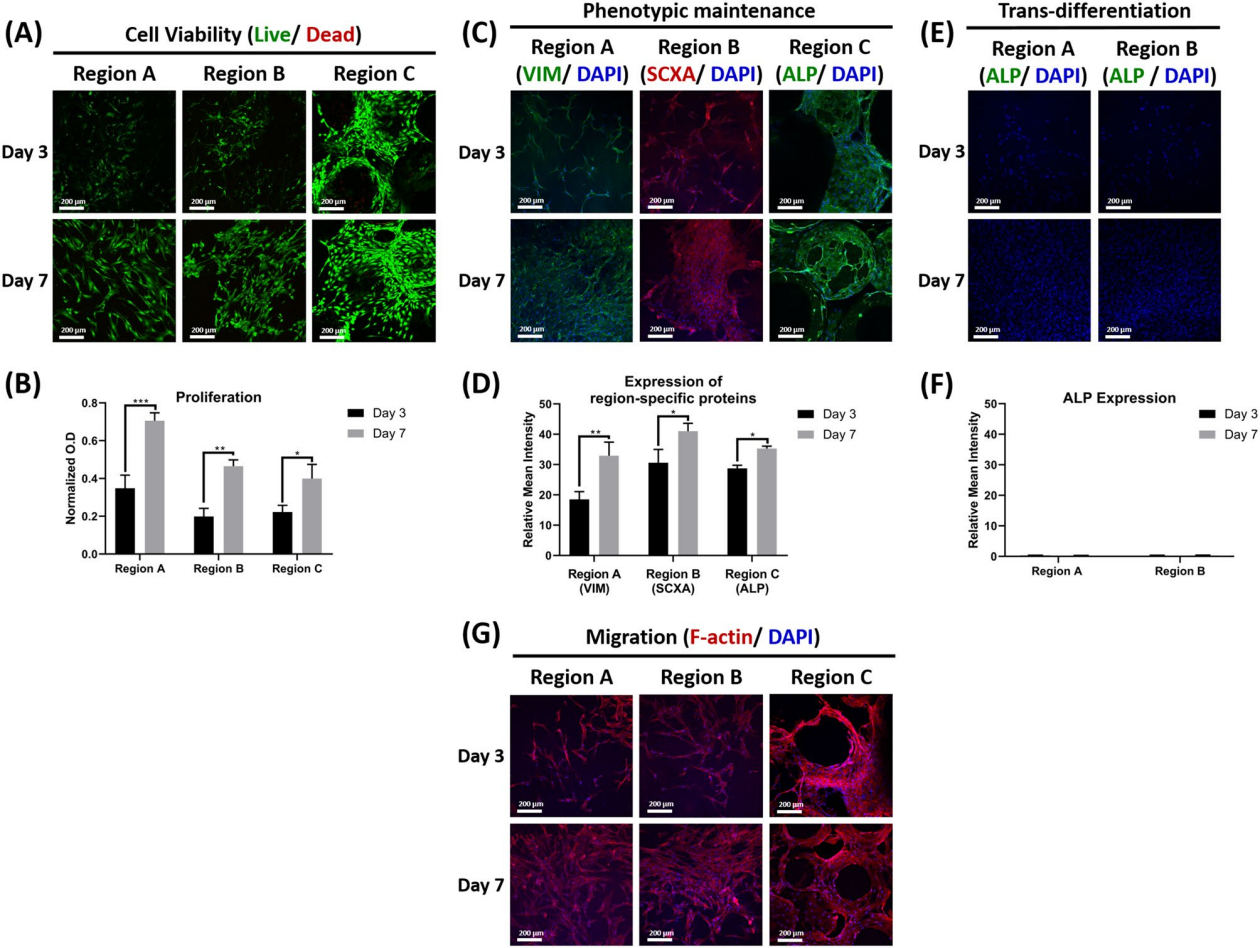
In vitro evaluations of the physiological functions and phenotypic characteristics in each region of the CGS during the heterogeneous culture. (**A**) Representative live/dead staining images for regions A, B, and C at 3 and 7 days (*n* = 3, and 4–6 random fields/sample). (**B**) Proliferation of SFs, STFs, and OBs in regions A, B and C, respectively at 3 and 7 days (*n* = 3, ANOVA and post hoc Tukey test, *P < 0.05, **P < 0.01, ***P < 0.001). (**C**) Immunofluorescent staining of regions A, B, and C for VIM, SCXA, and ALP at 3 and 7 days, respectively. (**D**) Quantifications of VIM, SCXA and ALP expression from regions A, B and C at 3 and 7 days, respectively (*n* = 3, and 4–6 random images/sample, ANOVA and post hoc Tukey test *P < 0.05, **P < 0.01). (**E**) Immunofluorescent staining of regions A and B for ALP at 3 and 7 days, respectively. (**F**) Quantifications of ALP expression from regions A and B at 3 and 7 days, respectively (*n* = 3, and 4–6 random images/sample, ANOVA and post hoc Tukey test). (**G**) Representative F-actin staining images for regions A, B, and C at 3 and 7 days (*n* = 3, and 4–6 random fields/sample).

Our data shows that the biomimetic structural cues provided within the CGS were able to maintain the different cells’ physiological functions and phenotypic characteristics during paracrine interactions, as they were able to maintain high viability, proliferation, migration, and phenotypic maintenance abilities at both time points as demonstrated by live/dead assay staining, MTS assay, actin staining, as well as immunofluorescent staining (Fig. 3).

Live/dead images showed that cells exhibited high viability at both time points (Fig. 3A). Proliferation assay showed that the three different cell types were able to proliferate within their respective regions and showed significantly higher proliferation at day 7 when compared to day 3 (Fig. 3B). Generally, fibroblasts at regions A and B showed higher proliferation when compared to OBs at region C at both time points, most likely because of the difference in proliferation rates between fibroblasts and osteoblasts^44^.

The three different cell types showed strong expression to the protein actin at both times as confirmed by the actin staining images (Fig. 3G). As cells substantially proliferated after 7 days, basal actin filaments were observed to be entirely interconnected and formed orthogonal networks on the surface of the different regions, indicating that the cells were able to fully spread and migrate to different parts within their respective regions. Although the pore volume at region C was filled with gelatin-mTG hydrogel, OBs were observed to have spread cell morphology throughout the region with robust cell migration at deeper regions, indicating that the presence of the hydrogel matrix did not hinder their spreading nor infiltration at this region.

Immunofluorescent images showed that cells in all regions were able to maintain their phenotype during the heterogeneous culture at both time points, as confirmed by the positive expression seen to their specific proteins (Fig. 3C). At day 7, all cells showed significantly higher expression to their specific proteins compared to day 3, indicating that these proteins were still able to be produced as cells were proliferating over time in the heterogeneous environment (Fig. 3D). Fibroblasts in both regions A and B were also immuno-stained with ALP to evaluate whether the paracrine factors mediated by OBs during the heterogeneous culture could induce their osteogenic differentiation. Both SFs and STFs in regions A and B did not show any expression to ALP at both time points (Fig. 3E,F) indicating that the paracrine factors mediated by OBs during the heterogeneous culture did not have stimulatory effects on both fibroblasts phenotypes, likely due to relevant biomimetic structural cues provided within the CGS which promoted their phenotypic maintenance.

Previous literature has shown hindered physiological functions and phenotypic characteristics of both SFs and STFs when co-cultured with OBs in monolayer-based co-culture systems^6–8^. The current study shows that combining the three different cell phenotypes in a physiologically relevant 3D microenvironment can rapidly maintain their relative physiological functions and phenotypic characteristics during the heterogeneous culture. These data imply the effectiveness of the CGS to act as a physiologically relevant milieu for studying different cell responses to heterogeneous culture. In addition, data indicate that the surrounding microenvironment plays a key role in modulating the cellular behavior during the heterogeneous culture and that conclusions derived from 2D monolayer-based culture systems are not reliable due to the lack of recapitulation of the tissue and organ-level structures and functions that are central for modulating biomimetic cellular responses.

### Achieving bioactive CGS by locally presenting different tissue-mediated growth factors in a region-specific manner

The feasibility of the CGS to act as a bioactive milieu by simultaneously enabling the presentation of different growth factors to achieve enhanced cell response while maintaining distinct cellular regions was investigated. This was achieved by incorporating different tissue-mediated growth factors in the three different regions. Specifically, ~ 1 µg of rhPDGF-BB, rhIGF-I, and rhBMP-2 were incorporated into regions A, B, and C, respectively. This resulted in a spatial and local presentation of growth factors throughout the CGS in a region-specific manner.

**Figure 4:**
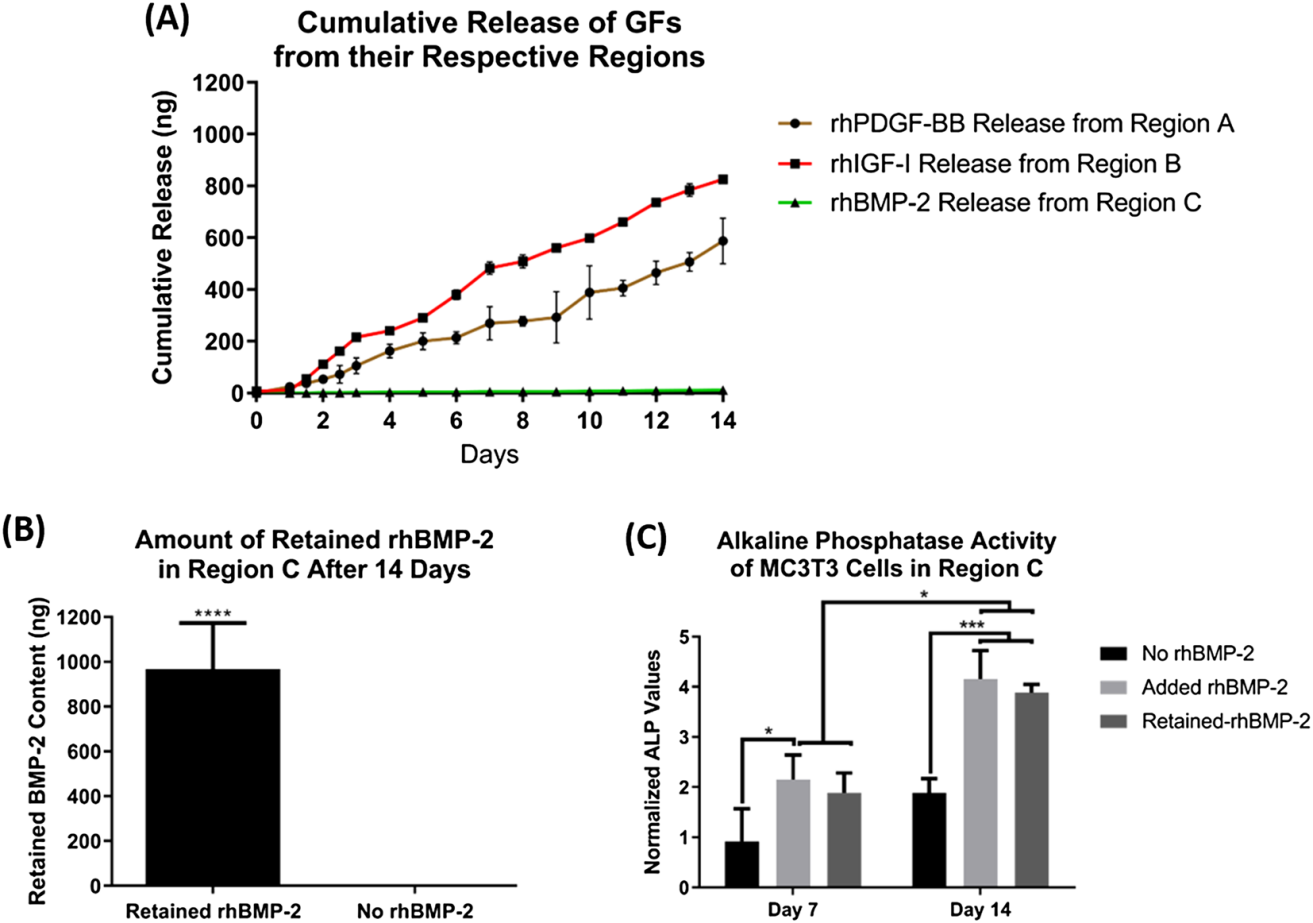
The cumulative release patterns of different growth factors incorporated into the different regions. Approximately 1 µg (1000 ng) of each growth factor was incorporated into the corresponding regions, followed by incubating them separately in PBS at 37 °C and monitoring their release patterns for 14 days (*n* = 5 per every region). (**A**) rhPDGF-BB, rhIGF-I and rhBMP-2 release patterns from regions A, B, and C, respectively. Coaxial nanofibers of both regions A and B could sustain and prolong the release of both rhPDGF-BB and rhIGF-I for 14 days. However, no evidence for rhBMP-2 release was observed during the 14 days study period, indicating the retention of rhBMP-2 in the gelatin-mTG hydrogel in region C. To confirm the retention of rhBMP-2 in region C the same samples used during the release study were incubated in collagenase solution to digest the hydrogel matrix for rhBMP-2 extraction. (**B**) Most of the protein was retained in all of the assayed samples with an average total retained protein of 967.5 ± 205 ng (*n* = 5, Student *t*-test, ****P < 0.0001). To evaluate the bioactivities of the retained rhBMP-2, MC3T3 cells were seeded on region C for 14 days and assayed for change in ALP activates. (**C**) The retained rhBMP-2 stimulated MC3T3 cells to secrete significantly higher levels of ALP after 14 days when compared to the negative control, indicating that rhBMP-2 is bioactive when retained (*n* = 3, ANOVA and post hoc Tukey test, *P < 0.05, ***P < 0.001).

Since the CGS consists of heterogeneous cell phenotypes, it was essential that the release of the incorporated factors from each region was tuned to avoid any potential undesirable effects that may occur due to improper singling. Therefore, the incorporation mechanism of the three growth factors into their respective regions (physical vs. immobilization) was determined based on their biological effects on the other cell phenotypes within the CGS. For instance, due to the mitogenic effects of both rhPDGF-BB and rhIGF-I on SFs, STFs, and OBs^45–50^, they were physically incorporated into the core–shell of the nanofibers of both regions A and B, respectively. This resulted in a sustained and prolonged release over the incorporated factors for 14 days (Fig. 4A), which suggests that they were locally being released within the domain of their respective regions. In contrast, due to the morphogenetic and osteoinductive effects of rhBMP-2 on both SFs and STFs^17,18,51^, it was immobilized by covalently binding it to the gelatin-mTG hydrogel matrix within region C to avoid its potential exposure to both fibroblasts phenotypes. Covalently binding rhBMP-2 resulted in its complete retention within region C, as no evidence for rhBMP-2 release was observed for 14 days (Fig. 4A). rhBMP-2 incorporated into the gelatin hydrogel was completely retained upon the addition of mTG due to the covalent bonding initiated between rhBMP-2 and gelatin as a result of the enzymatic crosslinking by mTG^40^.

To confirm the retention of rhBMP-2 the same samples used during the release study were subjected to digestion by incubation in collagenase type I solution to digest the gelatin-mTG matrix within region C in order to allow the retained protein to release, followed by measuring the amount of the released rhBMP-2 in the supernatant. The average total retained rhBMP-2 in all samples was found to be 967.5 ± 205 ng (Fig. 4B), indicating its successful retention.

To examine the bioactivity of the retained rhBMP-2 we measured the ALP activity of MC3T3 cells seeded on region C for 14 days and compared the results to region C-free of rhBMP-2 (negative control), and region C-free of rhBMP-2 with rhBMP-2 added to the culture medium (positive control). Data revealed that the MC3T3 cells showed significantly higher ALP levels at day 14 on both the experimental and the positive control groups when compared to the negative control, indicating that the retention of rhBMP-2 does not eliminate its bioactivity (Fig. 4C).

Previous studies have shown that growth factors covalently bonded in enzymatically crosslinked hydrogels only start releasing upon the degradation of the hydrogel matrix^52^. Because the release study here was carried out in phosphate-buffered saline (PBS), no release of the bonded rhBMP-2 was observed. Enzymatically crosslinked hydrogels only degrade when they are exposed to degrading enzymes such as collagenase or degrading enzymes endogenously secreted by cells^52^. When the retained rhBMP-2 in region C was examined for its bioactivity the ALP levels of the seeded MC3T3 cells were significantly elevated compared to the negative control at day 14. This suggests that the bonded rhBMP-2 was able to release and stimulate the cells due to the degradation of the gelatin-mTG matrix by the degrading enzymes endogenously secreted by the cells.

While surface immobilization has been shown to be an effective technique for localizing growth factors in a region-specific manner^21^, here, the physical incorporation of both rhPDGF-BB and rhIGF-I into regions A and B, as well as the retention of rhBMP-2 in the gelatin-mTG matrix within region C techniques were favored over immobilizing them at the surface of their respective regions for three reasons; (1) The amount of the loaded protein using the techniques used herein can be controlled compared to the surface immobilization technique, (2) The amount of the physically incorporated or retained protein is often larger than when they are surface-immobilized, as the amount of the surface-immobilized protein is limited by the surface area of the construct as well as the binding affinity/efficiency, and (3) Physically incorporated or retained proteins are continuously exposed to the cells for a prolonged period of time due to the continuous release of the loaded protein, whereas cells seeded on surface-immobilized protein based-construct are only limited to the amount of protein present on the surface of the construct that soon diminishes upon the subsequent degradation of the surface of the construct.

### Spatial localization of growth factors in a region-specific manner within the CGS resulted in heterogeneity maintenance and enhanced cell functions during a heterogeneous culture in vitro

**Figure 5:**
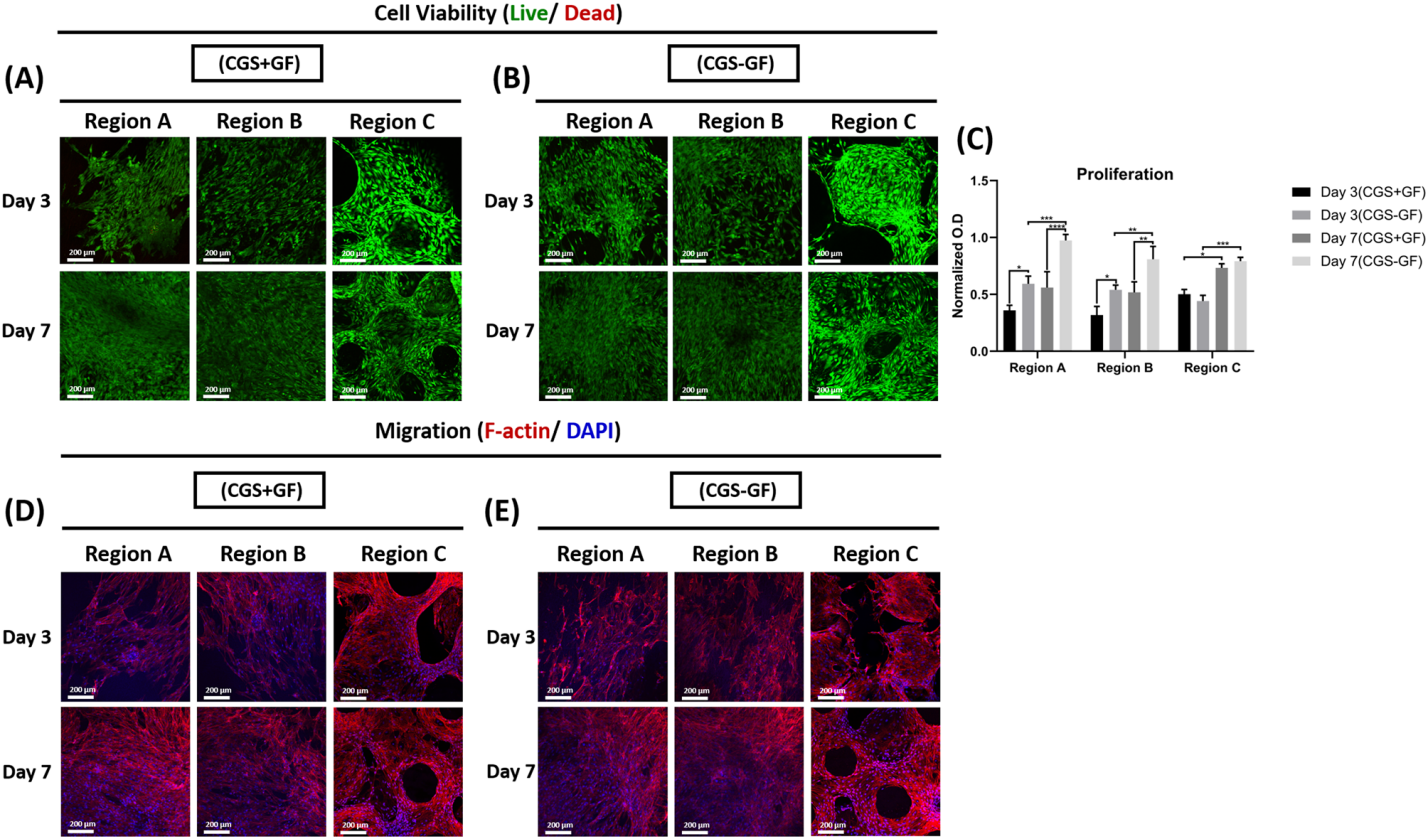
Effects of growth factors presentation mode on SFs, STFs and OBs viability, proliferation and migration during the heterogeneous culture. (**A**) Representative live/dead stained images for regions A, B and C in the CGS + GF and (**B**) CGS − GF groups at 3 and 7 days (*n* = 3, and 4–6 random fields/sample). (**C**) Proliferation of SFs, STFs, and OBs in regions A, B and C, respectively in the CGS + GF and CGS − GF groups at 3 and 7 days (*n* = 3, ANOVA and post hoc Tukey test, *P < 0.05, **P < 0.01, ***P < 0.001, ****P < 0.0001). (**D**) Representative F-actin staining images for regions A, B and C in the CGS + GF and (**E**) CGS − GF groups at 3 and 7 days (*n* = 3, and 4–6 random fields/sample).

The cells’ response to the growth factors addition into the CGS was evaluated in two different ways. 1) We first evaluated the role of the growth factors presentation mode on the different cells’ response during the heterogeneous culture. Here, growth factors were either specially localized into their respective regions within the graft system (CGS − GF), as discussed in the previous section or directly added to the growth medium during the culture time (CGS + GF) and the effects of the growth factors presentation mode on the physiological functions and the phenotypic characteristics of the different cell phenotypes in both groups were evaluated at 3 and 7 days. We found that in both groups the different cells maintained high viability at both time points (Fig. 5A, B) but proliferated differently (Fig. 5C). In the CGS + GF group, the proliferation of fibroblasts in both regions A and B was significantly suppressed at both time points compared to those in the CGS − GF group, which showed significantly higher proliferation. At day 7, fibroblasts in both regions A and B in the CGS − GF group showed significantly higher proliferation compared to day 3, but no statistically significant difference was found in those cultured in the CGS + GF between both time points. OBs in region C showed similar proliferation in both groups at days 3 and 7 with no statistically significant difference. At day 7, OBs in both groups showed significantly higher proliferation compared to their proliferation at day 3.

Interestingly, cells in all regions from both groups showed similar actin expression and filaments morphology at both time point as confirmed by the actin staining images with no significant differences (Fig. 5 D,E), indicating that cells in both groups were able to spread and migrate similarly within their respective regions regardless of the growth factors presentation mode.

**Figure 6:**
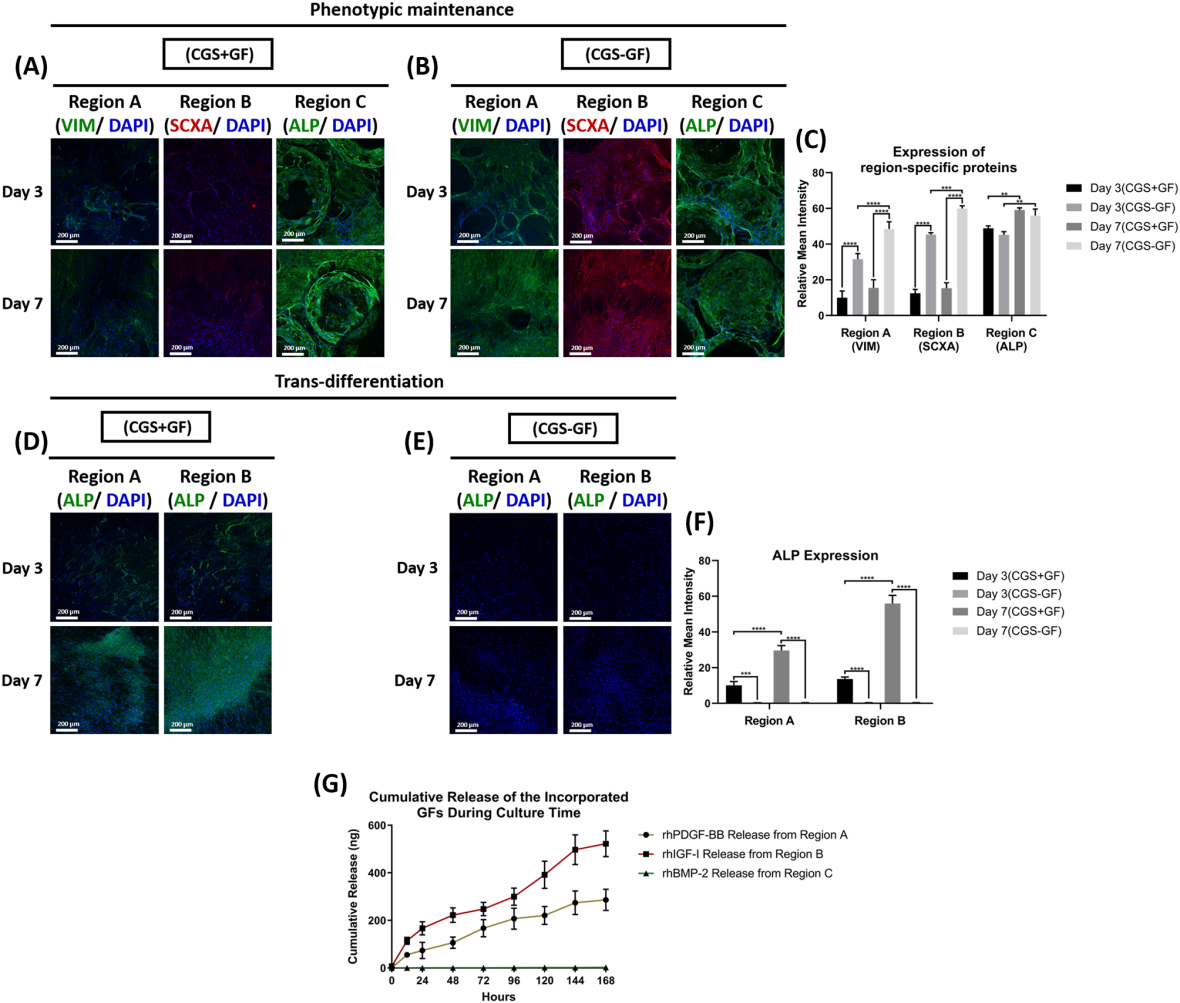
Effects of growth factor presentation mode on the phenotypic characteristics of SFs, STFs, and OBs during the heterogeneous culture. (**A**) Immunofluorescent staining of regions A, B, and C for VIM, SCXA, and ALP, respectively in the CGS + GF and (**B**) CGS − GF groups at 3 and 7 days and (**C**) quantifications of VIM, SCXA and ALP expression from regions A, B and C in the CGS + GF and CGS − GF groups at 3 and 7 days (*n* = 3, and 4–6 random images/sample, ANOVA and post hoc Tukey test **P < 0.01, ***P < 0.001, ****P < 0.0001). (**D**) Immunofluorescent staining of regions A and B for ALP in the CGS + GF and (**E**) CGS − GF groups at 3 and 7 days and (**F**) quantifications of ALP expression from regions A and B in the CGS + GF and CGS − GF groups at 3 and 7 days (*n* = 3, and 4–6 random images/sample, ANOVA and post hoc Tukey test ***P < 0.001, ****P < 0.0001). (**G**) The cumulative release of the incorporated growth factors from the CGS − GF group during 7 days of culture (*n* = 5).

Immunofluorescent images revealed that cells in regions A, B, and C in the CGS − GF group were able to maintain their phenotype as they were strongly positive for VIM, SCAX and ALP, respectively, at both time points (Fig. 6B) with a significant increase in expression at day 7 compared to day 3 (Fig. 6C). In contrast, only OBs in the CGS + GF group were able to show phenotypic maintenance ability at both time points with significantly higher ALP expression at day 7 compared to day 3 (Fig. 6A) and quantification data (Fig. 6C). There was no statistically significant difference in OBs ALP expression between CGS − GF and CGS + GF groups at both time points (Fig. 6C).

Both fibroblast phenotypes in regions A and B showed a significant reduction in the expression of their fibroblastic specific markers at both time points in the CGS + GF group compared to the CGS − GF group (Fig. 6A,B), with no statistical difference observed between days 3 and 7 (Fig. 6C). We attribute this to their direct exposure to rhBMP-2 upon its direct and uncontrolled presentation in this group, which may have caused trans-differentiation of both fibroblast phenotypes to an osteogenic lineage, owing to its osteoinductive effects^17,18,51^. This also explains the significant reduction seen in the proliferation of both fibroblast phenotypes in this group as cells tend to exit the division cycle as they undergo differentiation^53^.

To validate these findings, we evaluated the expression of ALP in both regions A and B by immunofluorescent staining at 3 and 7 days in both groups. Only SFs and STFs in the CGS + GF showed positive expression to ALP that significantly increased with time in comparison to the same cell phenotypes in the CGS − GF group, which showed no expression to ALP at both time points (Fig. 6D,E,F). To further confirm these findings, we measured the cumulative release of the incorporated growth factors in the CGS − GF group during the 7 days culture period. Measuring the cumulative release during the culture time was essential to confirm that rhBMP-2 was still completely retained within region C and that the endogenously secreted degrading enzymes by cells in region C are not causing it to be released to the culture medium. We found that only rhPDGF-BB and rhIGF-I released during the 7 days, while no evidence for rhBMP-2 release was observed (Fig. 6G), indicating its complete retention even during the culture time, and suggesting that the release of rhBMP-2 was within a limited domain, sufficient enough to only stimulate cells that are in close proximity or direct contact to the gelatin-mTG matrix within region C. This indicates that both fibroblast phenotypes were not being exposed to rhBMP-2 during the culture period, further elucidating the phenotypic maintenance of fibroblasts observed in this group in comparison to the CGS + GF group. Although both fibroblast phenotypes in the CGS − GF group were simultaneously being exposed to rhPDGF-BB and rhIGF-I during the culture period as evidenced by the release data (Fig. 6G), they were still able to maintain their relative heterogeneity. rhPDGF-BB and rhIGF-I are mitogenic factors that do not elicit any phenotypic modulatory effects on SFs or STFs ^45–48^. In fact, they both were shown to have synergetic effects in promoting the physiological functions of both SFs and STFs^47,48^. This further confirms that the only factor that caused the phenotypic alteration of both fibroblast phenotypes seen in the CGS + GF group was their direct exposure to rhBMP-2. In other words, if only rhPDGF-BB and rhIGF-I were to be added to the culture medium in the CGS + GF group, SFs and STFs would have been able to maintain their relative heterogeneity.

2) We next evaluated the effects of the growth factors themselves on the different cells’ performance in this system by making a direct comparison between the CGS and CGS − GF to investigate whether the spatial presentation of growth factors is enhancing the different cells’ performance during the heterogeneous culture. We found that not only the spatial presentation of growth factors within the graft system could maintain the different cells’ phenotype (Supplementary Fig. S8), but also significantly enhanced the region’s-specific protein expression (Supplementary Fig. S8A,B,C), viability (Supplementary Fig. S7A,B), and physiological functions such as proliferation and migration (Supplementary Fig. S7C,D,E), demonstrating the feasibility of the graft system to act as a bioactive milieu and its ability to control the presentation of different growth factors simultaneously for directing specific cell responses in a region-specific manner.

Collectively, these data provide evidence that CGS is a reliable tool that can be utilize for investigating different cell responses due to its closer resemblance to the native in vivo milieu. This includes but not limited to the investigation of the responses of distinct population of cells to paracrine interactions during a heterogenous culture. Moreover, data showed the feasibility of the CGS to act as a bioactive milieu and its ability to control the presentation of different growth factors simultaneously for directing specific cell responses by localizing the presentation of growth factors in a region-specific manner. We showed that the region-specific localization of growth factors, particularly those that elicit phenotypic modulatory effects on other cell phenotypes such as rhBMP-2, is crucial to enhance and maintain the different cells’ relative physiological functions and phenotypic characteristics during a heterogeneous culture in a single construct. This implies that the CGS presented herein could also be used as a platform for studying different approaches for simultaneously delivering multiple growth factors or molecules on a single construct to achieve enhanced cell response while maintaining cellular heterogeneity during heterogenous culture.

## Conclusion

In conclusion, a physiologically relevant 3D model that spatially modeled the 3D microenvironment of three main components of the limb, skin, soft tissue and bone was successfully developed. The model provided biomimetic structural cues and spatial presentation of tissue-mediated growth factors relevant to SFs, STFs, and OBs in a region-specific manner. The rationale for designing the CGS to accommodate these specific musculoskeletal cell types over others was due to the inability of both fibroblast phenotypes to maintain their relative physiological functions and phenotypic characteristics when co-cultured with osteoblasts due to the robust paracrine effects mediated by osteoblasts in co-culture. We showed that the biomimetic structural cues present within the model were alone sufficient in maintaining the heterogeneity of the three different cell phenotypes during a heterogeneous culture and paracrine interactions due to the close resemblance to the in vivo milieu, which helped regulating their relevant phenotypic characteristics. However, in the presence of growth factors, we showed that the structural cues have to be accompanied with controlled and local presentation of growth factors for avoiding improper signaling and achieving heterogeneity maintenance, as the biomimetic structural cues alone were not able to maintain distinct cellular regions when growth factors were presented in an uncontrolled manner to the cultured cells within the graft system. When growth factors were properly introduced to the cultured cells during heterogenous culture, enhanced physiological functions and phenotypic characteristics were observed. Our data suggest that providing biomimetic structural cues relevant to each cell phenotype and controlling the presentation of growth factors play a crucial role in ensuring heterogeneity maintenance of distinct cell populations during a heterogeneous culture. The presented CGS herein provides a reliable platform for investigating different cells responses to heterogeneous culture in a physiologically relevant microenvironment. In addition, the model provides a unique platform for evaluating the feasibility and efficacy of different approaches for simultaneously delivering multiple growth factors or molecules on a single construct to achieve enhanced cell response while maintaining cellular heterogeneity during a heterogenous culture. Current studies are investigating the in vivo efficacy of the CGS to support the simultaneous regeneration of these tissues and future work will focus on incorporating the muscle component into the CGS design.

## Materials and methods

### Fabrication of the three different regions within the CGS

Both regions A and B were fabricated by coaxial electrospinning. To do so, 16% (w/v) PLGA (85:15) (DURECT, Birmingham, AL, USA) (M_W_ ≈ 152 kDa) in 1,1,1,3,3,3-Hexafluoro-2-propanol (HFIP, Sigma-Aldrich, MO, USA) (shell solution), and 4% (w/v) type A gelatin (MP Biomedical, OH, USA) in HFIP alone or containing either rhPDGF-BB or rhIGF-I (Fisher Scientific, NH, USA) at a final concentration of 27 µg/mL (core–shell solution) were loaded into two separate 10 mL syringes and connected to a coaxial apparatus containing two concentric needles with different diameters, a 16 G (ID = 1.6 mm) outer needle and a 22 G (ID = 0.7 mm) inner needle. Each syringe was placed on a different electrospinning apparatus (NE 300 SYRINGE pumps, USA) and electrospun with varying flow rates, 1.5 mL/h for the shell solution and 0.75 mL/h for the core–shell solution at an applied voltage of 10 kV with the tip of the coaxial apparatus placed 10 cm away from the collector. The formed coaxial nanofibers were peeled from the collector, cut into 10 mm × 20 mm small scaffolds, and stored under a vacuumed desiccator for 24 h to ensure complete evaporation of solvent before use.

Region C was fabricated by heat sintering PLGA microspheres. Briefly, 12.5% (w/v) PLGA (85:15) in dichloromethane (Fisher Scientific) was poured into a 1% polyvinyl alcohol (Sigma-Aldrich) solution and was left to stir at 300 RPM overnight to form PLGA microspheres. Microspheres in the range of 300–600 µm were collected and packed into a cylindrical 10 mm × 10 mm stainless-steel mold and heat sintered at 90 °C for 90 min to construct region C. For rhBMP-2 (Fisher Scientific) incorporation into region C, 3% (w/v) type A gelatin in PBS (Gibco, NY, USA), and 10% (w/v) mTG (Ajinomoto, Japan) in PBS were sterilized through 0.22 µm filter and subsequently mixed at a ratio of 10:1 and stirred at 37 °C for 5 min to initiate gelatin gelation. Semi-gelled gelatin-mTG solution was mixed with rhBMP-2 at a final concentration of 10 µg/mL, and 100 µL of the produced mixture was injected into region C using an insulin syringe until the pore volume of the construct was filled, followed by incubation at 37 °C for 1 h to allow for a complete gelation of gelatin. For region C fabrication without rhBMP-2, gelatin-mTG solution was injected alone.

### Scanning electron microscopy (SEM)

All regions were morphologically evaluated under scanning electron microscopy post-fabrication using FEI Nova NanoSEM 450 (FEI Ltd., Tokyo, Japan) at a working distance of 5 mm and an acceleration voltage of 18 kV. Specimens were mounted on 15 mm stubs and were gold sputter-coated using (Polaron E5100) for 3 min to eliminate surface charging. Finally, the coated samples were loaded into the SEM and high magnification images of each region were taken.

### Particle size distribution and porosity analysis

Four to six different images from every sample/region (*n* = 4 per region) were obtained and analyzed for particle size distribution using Image J software (version 1.4 g, National Institute of Health, USA). To measure the porosity of every region (*n* = 4 per region), the following equation was used^54^:

$${\text{Region}}\;{\text{apparent}}\;{\text{density}}\,({\text{g/cm}}^{3} ) = \frac{{region\;Mass\,({\text{g}})}}{{region\;Volume \,({\text{cm}}^{3} )}}$$

$${\text{Region}}\,{\text{porosity}} = 1 - \frac{{region\,apparent\,density\,({\text{g/cm}}^{3} ) }}{{bulk\;density\;of\;PLGA\,({\text{g/cm}}^{3} ) }} \times 100\%$$

where PLGA bulk density is 1.27 (g/cm^3^).

### Transmission electron microscopy (TEM)

TEM analysis was conducted to confirm the coaxial structure of the nanofibers in both regions A and B. Observations were prepared by directly depositing the spun fibers onto copper grids of a 300 mesh. The samples for TEM images were analyzed using (FEI Tecnai 12 G2 Spirit BioTWIN) and all images were recorded in a GATAN ESW 500 camera.

### In vitro cumulative release of growth factors from different regions

The cumulative release of different growth factors incorporated into the different regions was measured for 14 days. Briefly**,** regions A, B, and C (*n* = 5 per each region) were placed in 5 mL glass vials filled with 2 mL of PBS and incubated at 37 °C under continuous agitation. At predetermined time points (0, 24, 36, 48, 60, 72 h and then every day for 11 days), 125 µL from every sample was collected and replaced with 125 µL of fresh PBS. Collected samples were stored at − 20 °C for later analysis. The cumulative release patterns of the different growth factors incorporated into the different regions were analyzed using the corresponding ELISA-kit (All from Fisher Scientific) to each protein following the manufacturer’s instructions. The initial protein content in both regions A and B was determined by a base-surfactant method^32^. Briefly, scaffolds of each region (*n* = 5) were subjected to hydrolysis in 0.1 N Sodium hydroxide (NaOH), 5 M Urea, 0.08% sodium dodecyl sulfate (SDS) in 50 mM Tris extraction medium at 37 °C for 3 h. After neutralization with 0.1 N Hydrochloric acid (HCL) and centrifugation, the protein concentration in the supernatant was measured using the corresponding ELISA-kits. For extracting the retained rhBMP-2 from gelatin-mTG in region C the same samples were incubated in 5 mL of 0.1% collagenase type I (ThermoFisher, MA, USA) solution in PBS at 37 °C until gelatin-mTG was digested, followed by measuring the protein content in the supernatant using corresponding ELISA-kits. For evaluating the bioactivity of the retained rhBMP-2 in gelatin-mTG in region C, MC3T3 cells (ATCC, VA, USA) were seeded on region C at a density of 1 × 10^5^ cells/sample and maintained at 37 °C, and 5% CO_2_ in 1 mL of MEM-α (1 ×) supplemented with 10% fetal bovine serum (FBS) and 1% Penicillin–Streptomycin 10,000U/mL (P/S) (Gibco). Region C-free of rhBMP-2 and region C-free of rhBMP-2 with 1 µg/mL of rhBMP-2 added to the culture medium served as negative and positive controls, respectively (*n* = 3 per every sample). At 7 and 14 days, ALP activity was measured using a BCA total protein kit (ThermoFisher) following the manufacturer’s instructions. Briefly, cells in region C were lysed by incubation in 1 mL of 0.1% Triton X-100 (Bio-Rad, CA, USA) through a freeze–thaw cycle and centrifugation. The cell lysate from every sample was used for the assay. ALP values were normalized to total protein values.

### Primary cell isolation and culture

All animal experiments were approved by the Institutional Animal Care and Use Committee (IACUC) at the University of Connecticut Health Center, CT, USA. All methods were performed in accordance with the relevant guidelines and regulations. Lewis rats (male, 6–8 weeks old) (Charles River Laboratories, MA, USA) were used for all primary cell isolations following previously established protocols with some modifications^55–57^ (Supplementary methods). SFs were maintained in culture in MEM supplemented with 10% FBS and 1% P/S. STFs were maintained in DMEM-F12 supplemented with 10% FBS, 1% P/S, and OBs were maintained in MEM-α (1 ×) supplemented with 10% FBS and 1% P/S (all from Gibco). All primary cells were used at the second passage for further experiments.

### CGS development and in vitro evaluations of region-specific heterogeneity maintenance

The CGS was cultured in three different conditions; (1) CGS (cells only), (2) CGS + GFs (cells and growth factors directly supplemented in medium) and (3) CGS − GFs (cells and growth factors incorporated into their designated regions). Developing the CGS was achieved through multiple steps. First, all regions were sterilized prior to cell seeding. Regions A and B containing growth factors or free of growth factors were sterilized by direct exposure to ultraviolet (UV) light for 30 min per side. Region C was sterilized by immersion in 70% ethanol for 30 min, followed by immersion in PBS for 30 min and then exposed to UV light for 30 min per side. Gelatin-mTG solution containing rhBMP-2 or free of rhBMP-2 was incorporated into region C after sterilization. Regions A, B, and C were placed separately in low-binding 24-well plates (*n* = 3/region/condition) and seeded with SFs, STFs, and OBs, respectively, at a density of 1 × 10^5^ cells/region. Cells were allowed to partially attach for 1.5 h at 37 °C. Next, 1 mL of MEM-α (1 ×) supplemented with 10% FBS and 1% P/S alone or premixed with the corresponding growth factors at a concentration of 1 µg/mL was added to each region and maintained in an incubator at 37 °C, and 5% CO_2_ for 2 days to allow the cells to completely adhere to their respective regions_._ At day 2, all regions were harvested and brought together to construct the CGS in which region B was wrapped around region C, followed by wrapping region A around both regions B and C. The regions were then tied using 5–0 vicryl sutures (Ethicon. Inc, NJ, USA) to keep them in place and placed in fresh low-binding 24-well plates. One mL of the medium was added to both conditions 1 and 3 or premixed (condition 2) with the three different growth factors at a concentration of 1 µg/mL per growth factor and further incubated. Three and 7 days post-CGS development, the CGS was harvested, regions were separated, washed twice with PBS and assayed for cell viability, proliferation, migration, and phenotypic maintenance.

For cell viability evaluations, live/dead assay/cytotoxicity kit (Invitrogen, CA, USA) was used following the manufacturer’s instructions. Briefly, every region was incubated in 1 mL of the assay solution (2 µL/mL EthD-1, and 0.5 µL/mL calcein-AM in PBS) for 1 h at room temperature (RT) in the dark. Next, regions were washed twice with PBS and 4–6 random images per each sample were taken.

For proliferation evaluations, an MTS assay (MTS, Promega Inc, WI, USA) was performed following the manufacturer’s instructions. Every region was incubated in 1 mL of a cocktail solution of the MTS reagent premixed with the medium at a ratio of (1:5) for 3 h at 37 °C in the dark. Next, 100 uL from each sample was collected and transferred to a 96-well plate and the absorbance was measured at 490 nm with a spectrophotometer (TECAN, Crailsheim, Germany).

For migration evaluations, the cells’ cytoskeletal protein, actin, was stained by rhodamine-phalloidin staining. Regions were fixed in 4% paraformaldehyde (Sigma-Aldrich) in PBS for 20 min, permeabilized with 0.1% Triton X-100 for 10 min, and blocked with 1% bovine serum albumin (BSA, Sigma-Aldrich) for 30 min. Regions were then stained with F-actin Alexa Fluor 594-conjugated (1:50) (Invitrogen) for 60 min in the dark, followed by nuclei staining with 4′, 6-Diamidino-2-Phenylindole, Dihydrochloride (DAPI, Invitrogen) at a concentration of 1:3000 for 10 min. Regions were then washed twice with PBS and 4–6 random images per sample were taken. Staining was performed at RT.

For phenotypic maintenance evaluations, immunofluorescent staining for vimentin (VIM), scleraxis (SCXA) and alkaline phosphatase (ALP) was performed. Regions were fixed with 4% paraformaldehyde in PBS for 20 min, permeabilized with 0.1% Triton X-100 for 10 min and blocked with 10% goat serum (Gibco) in PBS for 1 h. Region A was then incubated with anti-vimentin (ab92547, 1:500 Abcam, Cambridge, UK) or anti-ALP (ab218574, 1:100, Abcam), region B was incubated with anti-SCXA (ab58655, 1:1000, Abcam) or anti-ALP, and region C was incubated with anti-ALP in the same blocking buffer for 2 h. Following, regions were washed twice with PBS and incubated with the secondary antibodies goat anti-rabbit Alexa Fluor 488 (ab150077, 1:500, Abcam) or goat anti-rabbit Alexa Fluor 594 (ab150080, 1:500, Abcam) for 2 h in the dark, followed by washing twice with PBS and incubation in DAPI (1:3,000) for 10 min. Regions were then washed twice with PBS and 4–6 random images per each sample were taken. Staining was performed at RT. All samples for cell viability, migration, and phenotypic maintenance evaluations were visualized using an inverted fluorescence microscope (Zeiss LSM 880, Oberkochen, Germany) using Zen Software. Protein expression in all images was quantified by measuring the mean intensity values using ImageJ software (*n* = 3 and 4 – 6 random fields per sample).

### In vitro cumulative release of different growth factors incorporated into the CGS during culture

The cumulative release of the different growth factors incorporated into the CGS was measured for 7 days during culture. Briefly, the CGS was sterilized, populated with cells, developed, and cultured as described previously (*n* = 5). At predetermined time points (0, 12, and 24 h, and then every day for 6 days), 75 µL from every sample was collected and replaced with 75 µL of fresh medium. Collected samples were stored at − 20 °C for later analyses. The cumulative release of the different growth factors was analyzed using the corresponding ELISA-kit to each protein following the manufacturer’s instructions. Routine medium change was not done as to maintain constant protein levels during the release study.

### Statistical analysis

Data are presented as mean ± standard deviation (SD). All statistical analyses were performed using the statistical software Prism GraphPad version 8 (GraphPad, San Diego, CA). One or where appropriate two-ways analysis of variance (ANOVA), Tukey post hoc testing and Student *t*-tests were applied to mean comparisons. The difference between experimental groups was considered statistically significant at a *P* value of less than 0.05.

## Supplementary information

Supplementary Information 1.

Supplementary Information 2.

Supplementary Information 3.

## Data Availability

The datasets generated and/or analyzed during the current study are available from the corresponding author upon reasonable request.
