# Supplementary material for: Robust phenotypic maintenance of limb cells during heterogeneous culture in a physiologically relevant polymeric-based constructed graft system
Source: Sci Rep. 2020 Jul 16;10:11739. doi: 10.1038/s41598-020-68658-z (PMC7367281; doi:10.1038/s41598-020-68658-z)
Supplement: Supplementary file 1 — Supplementary Information 1. [file 41598_2020_68658_MOESM1_ESM.docx]

**Supplementary Information**

**Robust Phenotypic Maintenance of Limb Cells during Heterogeneous Culture in a Physiologically Relevant Polymeric-Based Constructed Graft System**

**Mohammed A. Barajaa^1,2^, Lakshmi S. Nair^1,2,3,4,5,6,7^, Cato T. Laurencin^1,2,3,4,5,6,7,8*^**

**^1^**Connecticut Convergence Institute for Translation in Regenerative Engineering, University of Connecticut Health Center, Farmington, CT, 06030, USA;

**^2^**Department of Biomedical Engineering, University of Connecticut, Storrs, CT, 06269, USA;

**^3^**Raymond & Beverly Sackler Center for Biomedical, Biological, Physical & Engineering Sciences, University of Connecticut Health Center, Farmington, CT, 06030, USA;

**^4^**Department of Orthopedic Surgery, University of Connecticut Health Center, Farmington, CT, 06030, USA;

**^5^**Department of Materials Science & Engineering, University of Connecticut, Storrs, CT, 06269, USA;

**^6^**Institute of Materials Science, University of Connecticut, Storrs, CT, 06269, USA;

**^7^**Department of Chemical & Biomolecular Engineering, University of Connecticut, Storrs, CT, 06269, USA;

**^8^**Department of Craniofacial Sciences, School of Dental Medicine, University of Connecticut Health Center, Farmington, CT, 06030, USA.

**Supplementary Methods**

**Primary cell isolation and culture.**

6-8 weeks old (150 – 200 g) Lewis rats (Charles River Laboratories, MA, USA) were used for all primary cell isolations following previously established protocols with some modifications^54–56^. All animal experiments were approved by the Institutional Animal Care and Use Committee (IACUC) at the University of Connecticut Health Center, CT, USA. All methods were performed in accordance with the relevant guidelines and regulations. For SFs isolation and culture approximately a 1 cm^2^ skin fragment from the animal’s underarm was collected, rinsed in sterile PBS containing 1% Antibiotic-Antimycotic (A-A, Gibco), and minced into less than 1 × 1 mm^2^ following the removal of fat tissue and blood vessels. The minced skin was digested in 30 mL DMEM/F12 containing 1% antibiotic-antimycotic and 0.14 Wunsch units/mL liberase blendzyme 3 (Sigma-Aldrich) and stirred for 2 hours at 37°C. Next, 20 mL of DMEM/F-12 supplemented with 15% FBS and 1% A-A was added to the digested skin to stop liberase digestion. The skin fragments were transferred into a 50 mL conical tube and centrifuged at 524 g for 5 minutes (repeated 3 times (3X)). Following centrifugation, the supernatant was removed and the pellet was re-suspended with 10 mL of medium and transferred into a 10 cm tissue culture plate (TCP) and placed in an incubator at 37°C and 5% CO_2_ for 14 days to allow the cells to completely exit the tissue fragments and adhere to the TCP. Seven days post-initial plating the medium was changed and the plate was incubated for an additional 7 days. Fourteen days post-initial plating (~ 90% confluency), the cells were detached using 0.25% Trypsin-EDTA (Gibco) and expanded in MEM supplemented with 10% FBS, 1% P/S.

For STFs isolation and culture, anterior cruciate ligaments (ACL) from both knees were collected, rinsed in sterile PBS containing 1% P/S and minced into approximately 1 × 1 mm^2^ pieces. The minced ACL tissues were digested in 30 mL DMEM/F-12 containing 1% A-A and 0.15% type I collagenase and stirred for 4 hours at 37°C. Next, 20 mL of DMEM/F-12 supplemented with 10% FBS and 1% A-A was added to the solution to stop the collagenase digestion, followed by passing the solution through a 70 µm cell strainer then centrifugation at 500 g for 10 minutes. Following centrifugation, the supernatant was removed and the pellet was re-suspended with 5 mL of medium, transferred into a 25 cm^2^ flask, and placed in a tissue culture incubator at 37°C and 5% CO_2_. The medium was changed every 2 and 7 days post-initial plating. At ~ 90% confluency, the cells were detached using 0.25% Trypsin-EDTA, and expanded in DMEM-F12 supplemented with 10% FBS, 1% P/S.

For OBs isolation and culture, femurs from both limbs were collected, rinsed in sterile PBS containing 1% P/S, followed by scraping the surface of the bone to remove residual muscle and connective tissues. Next, the bone epiphyses were cut off and the bone marrow was flushed with PBS until the bone cavity appeared pale. OBs were obtained from multiple digestion cycles. **Digest cycle 1**: paled femurs were cut into 1 – 2 mm^2^ small pieces and incubated in 8 mL hank’s balanced salt solution (HBSS, Gibco) containing 0.1% type I collagenase by vortexing for 1 hour at 37°C. The digestion solution was transferred into a 50 mL conical tube and the bone pieces were washed 3X with 5 mL of fresh HBSS in the same conical tube containing the digestion solution, followed by centrifugation at 500 g for 5 minutes. Following centrifugation, the supernatant was removed and the pellet was re-suspended with 5 mL MEM-α (1X) supplemented with 10% FBS and 1% P/S, transferred into a 25 cm^2^ flask and incubated at 37°C, 5% CO_2_. For obtaining OBs from **digest cycles 2 and 3**, the same procedure as described in digest 1 was performed using the same bone pieces from digest 1. The medium was changed every 3 and 7 days post-initial plating. At ~ 90% confluency, the cells were detached using 0.25% Trypsin-EDTA and expanded in the same medium. All primary cells were used at the second passage for further experiments.

**Immunofluorescence phenotypic characterization of isolated primary cells.**

The three different cell types were directly seeded on coverslips (*n* = 4 per cell type) in 12-well plates at a density of 3 × 10^4^ cells/ mL and cultured for 3 days in the corresponding growth medium as described above. After 3 days, the medium was aspirated and cells were washed twice with PBS. Next, cells were fixed with 4% paraformaldehyde in PBS for 20 minutes, permeabilized with 0.1% Triton X-100 for 10 minutes and blocked with 10% goat serum in PBS for 1 hour. Next, SFs were incubated with anti-vimentin (ab92547, 1:500 Abcam) or anti-SCXA (ab58655, 1:1000, Abcam) (negative control), STFs were incubated with anti-SCXA or anti-vimentin (negative control) and OBs were incubated with anti-ALP (ab218574, 1:100, Abcam) or anti-SCXA (negative control) in the same blocking buffer for 2 hours. Cells were washed twice with PBS and incubated with the secondary antibodies; goat anti-rabbit Alexa Fluor 488 (ab150077, 1:500, Abcam) or goat anti-rabbit Alexa Fluor 594 (ab150080, 1:500, Abcam) for 2 hours in the dark, followed by washing twice with PBS and incubation in DAPI (1:3000) for 10 minutes. Cells were then washed twice with PBS and 4-6 random images per each sample were taken. Staining was completed at RT. Cells were visualized using an inverted fluorescence microscope (Zeiss LSM 880, Oberkochen, Germany) using Zen Software. For quantifying the positive expression for every antibody, the number of positive cells was divided by the number of nuclei (DAPI) and multiplied by 100 using ImageJ software (4-6 images per antibody).

**Development of the Tri-culture system.**

**Mold design, development, and three-dimensional printing.**

A mold was designed and 3D printed in order to be used for fabricating the polydimethylsiloxane (PDMS, Dow Corning, MI, USA) inserts. The mold consisted of two parts, a disc and a plate that served as a housing unit for the disk. The two parts were first 3D designed using SolidWorks 3D CAD software. The disc had a diameter of 17 mm and a thickness of 5 mm with an internal gap distance of 2 mm. The plate had a length of 13 mm, a width of 8 mm and a height of 2 mm with an internal hole diameter of 17 mm. Both parts were 3D printed using the MakerBot Replicator (Makerbot, USA). Poly (lactic acid) (PLA, Dell, TX, USA) filaments were loaded into the 3D printer syringe and heated to 50°C to melt the polymer. Both parts were 3D printed with a syringe nozzle of 200 µm and a printing velocity of 5 mm/s. Parts were then harvested and stored.

**Polydimethylsiloxane (PDMS) insert fabrication**

PDMS elastomer was mixed with a curing agent at a ratio of 10:1 elastomer to curing agent to fabricate the PDMS inserts. The viscous solution was mixed thoroughly for 5 minutes to ensure homogeneity between the elastomer and the curing agent. The mixture was then placed in a dissector until all bubbles were removed. To fabricate the PDMS inserts the 3D printed disc was inserted into the holes within the plate, followed by pouring the PDMS elastomer into the three-star pointed shaped mold cavity. The mold containing PDMS elastomer was then incubated at 37°C for 24 hours so that the PDMS elastomer could completely cure. The PDMS inserts were then removed from the mold and stored in a dissector for later use.

**Validation of the Tri-Culture System.**

Validation of the tri-culture system was accomplished in two steps:

**Liquid penetration**

PDMS inserts were inserted into the bottom of the wells of a 24-well plate, generating three equal chambers. Next, 200 µl of PBS was added to the first chamber, 200 µl of a red dye was added to the second chamber, and 200 µL of a blue dye was added to the third chamber within the same well. The plates were incubated at 37°C under gentle agitation for 3 and 7 days (*n* = 3/ time point). At 3 and 7 days, the penetration of the three colored dyes to the other chambers was qualitatively examined.

**Cell migration**

Prior to seeding cells into the different chambers, the three different cell types were fluorescently labeled according to the cell type. Cells were separately seeded in 10 cm tissue culture plates at a density of 1 × 10^6^ cells/ mL and cultured in the corresponding mediums for 24 hours to allow for cell adherence. Next, the corresponding mediums were removed and replaced with a cocktail of mediums premixed with lipophilic dye agents at a concentration of 8 µg/ mL. Specifically, SFs were labeled with IncuCyte® NucLight Orange Lentivirus Reagent, STFs were labeled with IncuCyte® NucLight Red Lentivirus Reagent, and OBs were labeled with IncuCyte® NucLight Green Lentivirus Reagent (Essen BioScience Inc, MI, USA). Cells were exposed to the dye agents for 24 hours, followed by washing with PBS and further cultured in the corresponding mediums until the desired confluency was obtained (~ 90% confluency). Next, the labeled cells were detached using 0.25% trypsin EDTA and a 200 µL cell suspension of every cell type was added to every chamber at a concentration of 25 × 10^3^/ mL (5k cells/ chamber/ cell type) (*n* = 3/ time point). At 3 and 7 days, mediums were aspirated; PDMS inserts were removed, PBS was added to each well and cellular migration to other chambers was assessed using an inverted fluorescence microscope (Leica DMi8) using LAS X software.

***In vitro* optimization of an optimal growth medium for a heterogeneous culture.**

To optimize an optimal growth medium for a heterogeneous culture, the different cell phenotypes were cultured either alone (single culture environment (SCE)) or together (tri-culture environment (TCE)) in different types of mediums; (1) SFs medium [MEM supplemented with 10% FBS, 1% P/S], (2) STFs medium [DMEM: F-12 supplemented with 10% FBS and 1% P/S, (3) OBs medium [MEM - α (1×) supplemented with 10% FBS and 1% P/S], (4) [2:1:1], (5) [1:2:1], (6) [1:1:2], and (7) [1:1:1], respectively (*n* = 3 for every cell phenotype/ medium type/ condition). For culturing the cells in the TCE, autoclaved PDMS inserts were introduced into the bottom of the 24-well plates. Next, a 200 µL cell suspension of every cell type in the corresponding medium was added to every chamber at a concentration of 25 × 10^3^/ mL (5k cells/ chamber/ cell type). For culturing the cells in the SCE the same procedure was completed expect for that a single chamber of every well was only occupied by a single cell population. Plates were incubated at 37°C and 5% CO_2_ for 24 hours to allow cells to completely adhere to their respective chambers, followed by the addition of 400 µl (800 µl in the case of SCE) of the corresponding medium type to each well to bring the total volume to 1 mL/ well. Mediums were changed every 3 days. The growth of the cells in both conditions, the SCE and TCE, was examined using an MTS assay kit following the manufacturer’s instructions. At predetermined time points, 3 and 7 days, mediums were removed and cells were washed with PBS. A 200 µL cocktail solution of MTS reagent premixed with the corresponding medium type at a ratio of (1:5) was added to every chamber, followed by incubation for 3 hours at 37°C in the dark. Next, 100 µL from each chamber was collected and transferred to a 96-well plate and the absorbance was measured at 490 nm. Data were presented as the average increase in the fold between day 3 and day 7 of three experimental replicates for every cell type cultured in every medium in each condition.

**Supplementary Results:**


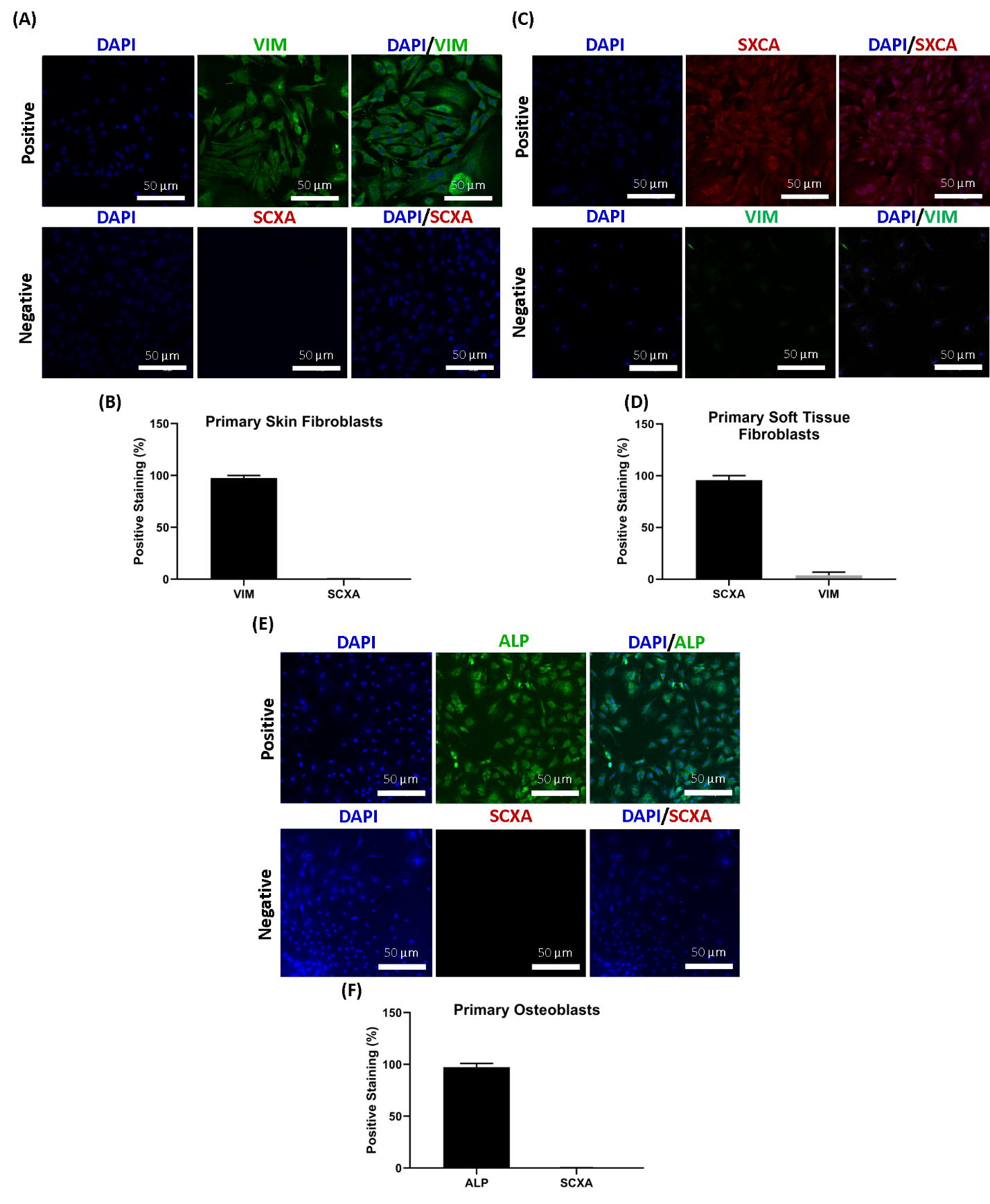


**Fig. S1:** Immunofluorescent phenotypic characterization of isolated primary cells and their corresponding positive staining quantifications. **(A-F)** All isolated cells were positive for their corresponding markers and negative for the controls, indicating the homogeneity in the isolated cellular population. STFs showed a slight expression to the SFs specific marker VIM, indicating that it also can be expressed in STFs but in lower amounts (*n* = 4/ marker)


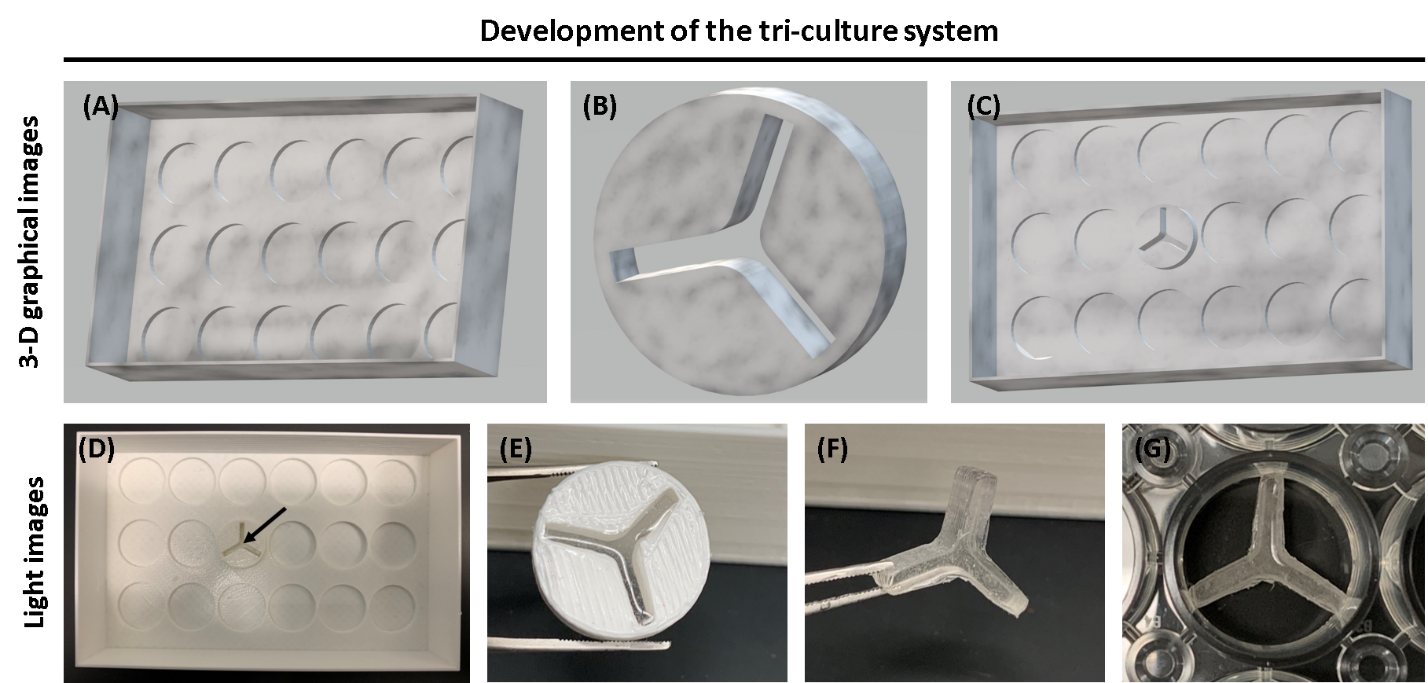


**Fig. S2: 3D graphical and light images demonstrating the various steps involved in the development of the tri-culture system**. **(A)** A 3D view showing the plate, and **(B)** the disc that were used to fabricate the PDMS inserts. **(C)** The plate served as a platform that housed the disc in the provided holes to prevent the PDMS from leaking from the sides of the disc during the pouring process of the elastomer into the mold. **(D)** The plate and the disc were 3D printed, and the disc was inserted into the provided holes within the plate, filled with PDMS elastomer and incubated at 37°C for 24 hours to allow the PDMS to completely cure. **(E)** After they cured, **(F)** the PDMS inserts were removed from the mold and **(G)** placed inside of a 24-well plate. Placing the PDMS inserts into the well separated the well into three different chambers with symmetrical volumes in which each chamber could be used to culture a different cell population. Black arrow indicates the cavity within the disc in which the PDMS elastomer was poured to form the three-points star-shaped PDMS insert.


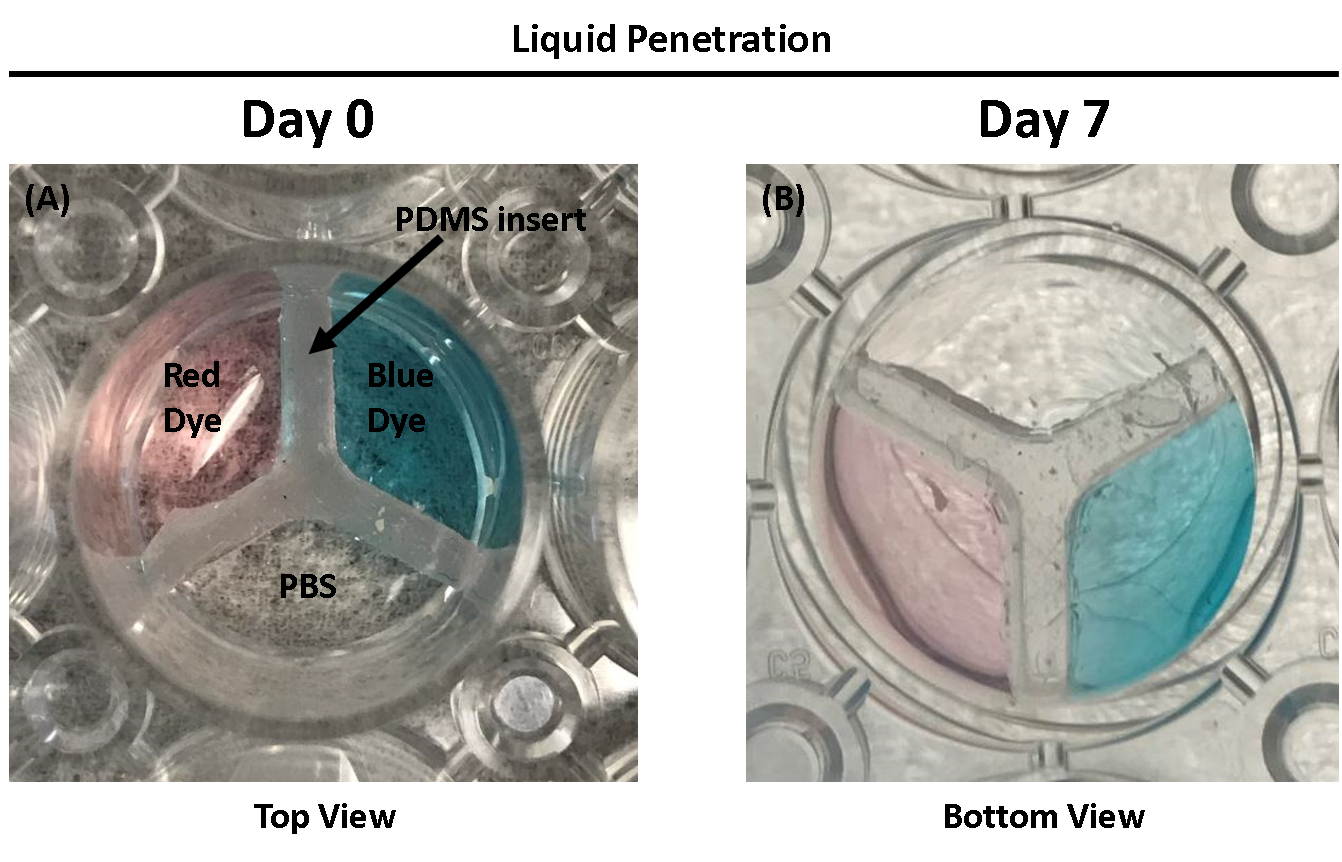


**Fig. S3:** **Validation of the tri-culture system by liquid penetration**. To validate the functionality of the tri-culture system, **(A)** PDMS inserts were inserted into the 24-well plate and the generated chambers were filled with different colored dyes, red, blue and transparent (PBS)**,** and incubated at 37°C for 7 days on a rotatory rocker. **(B)** 7 days post-incubation, all dyes were shown to be well contained within the chambers they were initially added in and no liquid penetration to the different chambers was observed indicating that the PDMS insert provided a physical barrier between the different chambers.


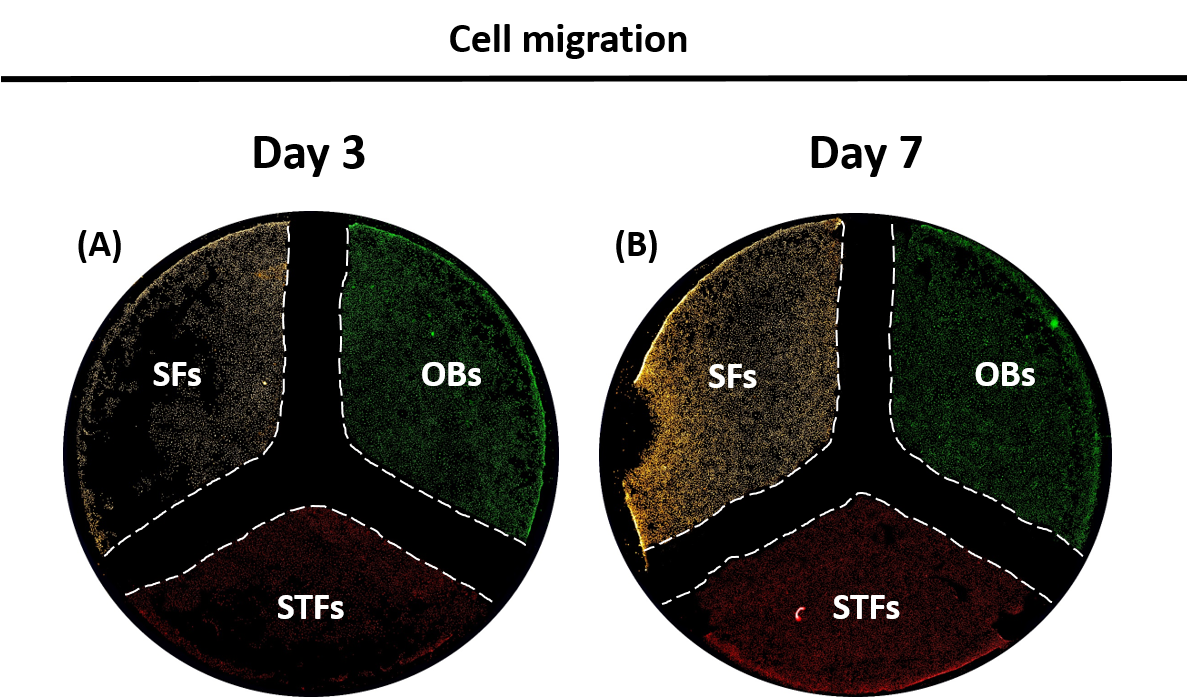


**Fig. S4:** **Validation of the tri-culture system by cell migration.** To further validate the functionality of the tri-culture system, PDMS inserts were inserted into the 24-well plate and the generated chambers were seeded with fluorescently labeled SFs, STFs, and OBs and incubated at 37°C for **(A)** 3 and **(B)** 7 days. All three cell types were well maintained in their respective chambers and no cellular migration to other chambers was observed at all time points, which further indicates the physical separation provided due to the insertion of the PDMS insert, which acted as a physical barrier between the different cell phenotypes.

**
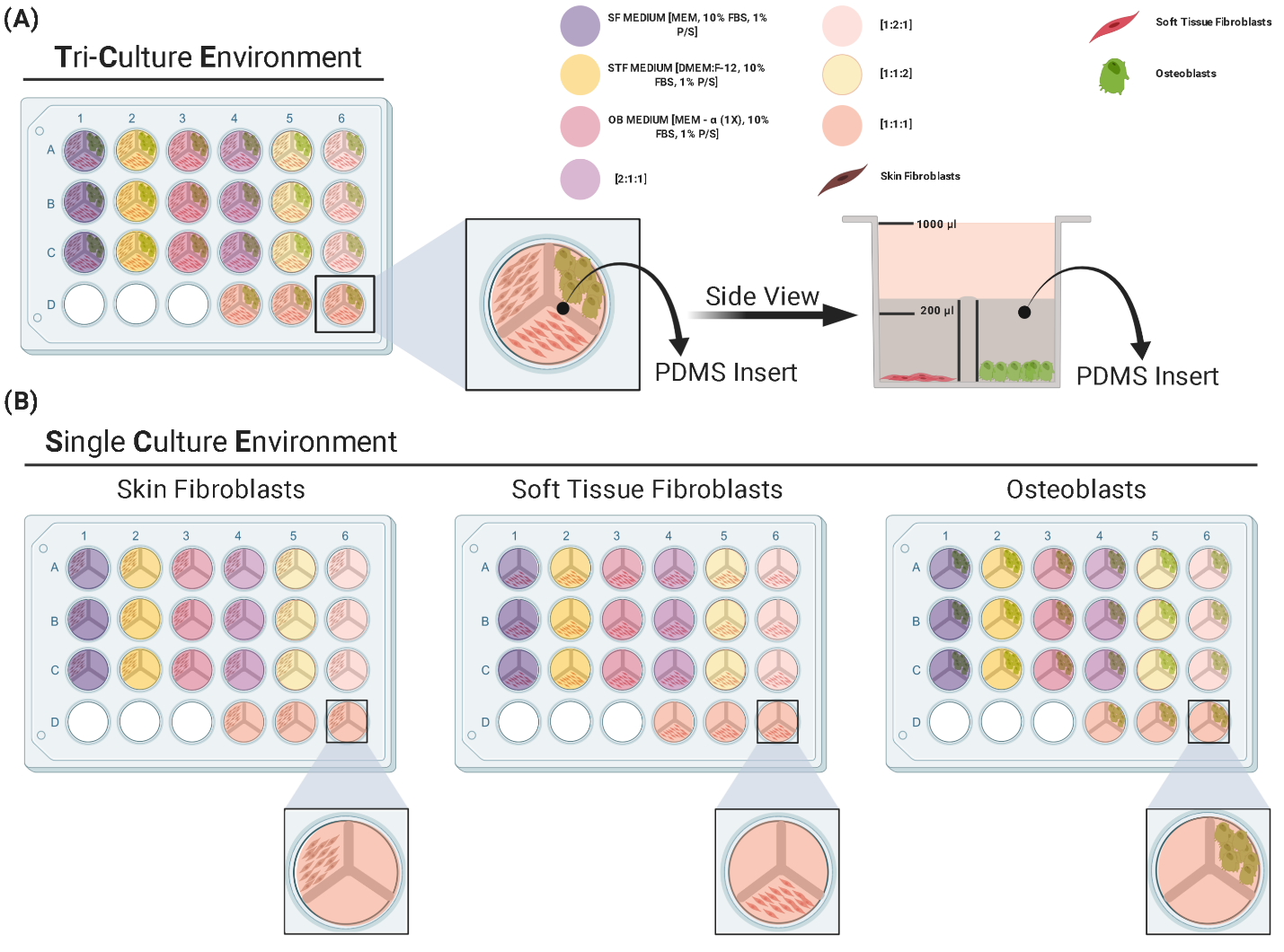
**

**Fig. S5:** Representative schematic demonstrating the experimental setup for the determination of an optimal heterogeneous growth medium for the three different cell types in **(A)** condition 1: Tri-culture environment (TCE) and **(B)** condition 2: single culture environment (SCE)


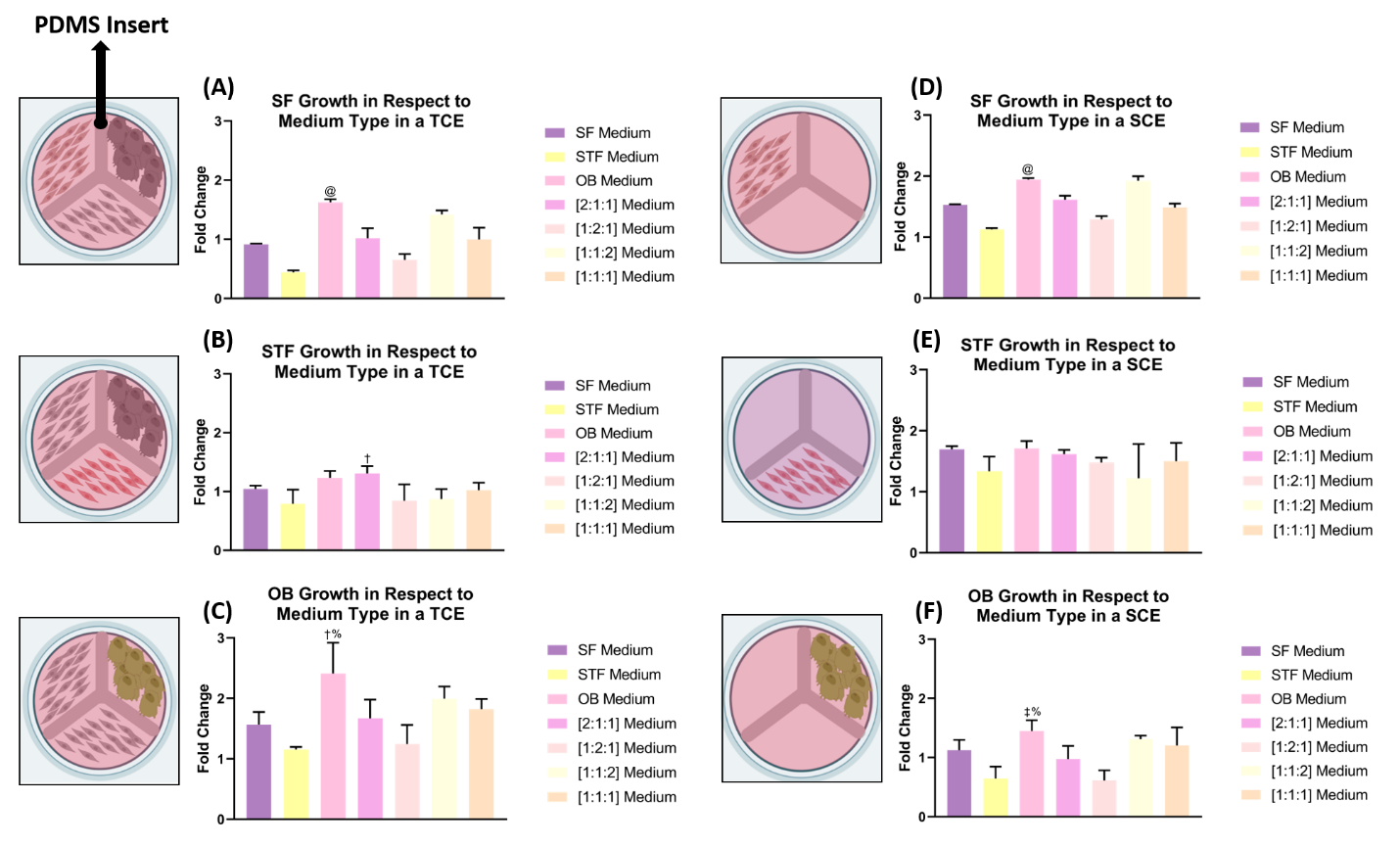


**Fig. S6:** **The growth of SFs, STFs, and OBs in respect to medium type in a TCE and SCE (Graphical representation of data).** **(A)** The growth of SFs in different types of medium in a TCE (*n* = 3; ANOVA and *post hoc* Tukey test, ^@^P < 0.0001 compared to all mediums except for [1:1:2] P = 0.1589), **(B)** the growth of STFs in different types of medium in a TCE (*n* = 3; ANOVA and *post hoc* Tukey test, ^†^P < 0.05 compared to STFs, [1:2:1] and [1:1:2] mediums) and **(C)** the growth of OBs in different types of medium in a TCE (*n* = 3; ANOVA and *post hoc* Tukey test, ^†^P < 0.05 compared to SFs and [2:1:1] mediums, ^%^P < 0.001 compared to STFs and [1:2:1] mediums. **(D)** The growth of SFs in different types of medium in a SCE (*n* = 3; ANOVA and *post hoc* Tukey test, ^@^P < 0.0001 compared to all mediums except for [1:1:2] P = 0.9919), **(E)** the growth of STFs in different types of medium in a SCE (*n* = 3; ANOVA and *post hoc* Tukey test, no significance when compared to other medium types), and **(F)** the growth of OBs in different types of medium in a SCE (*n* = 3; ANOVA and *post hoc* Tukey test, ^‡^P < 0.01 compared to STFs medium, ^%^P < 0.001 compared to [1:2:1] medium). Results indicate that the OBs medium supported the growth of the three different cell types in both conditions, the TCE and SCE, suggesting its suitability as an optimal heterogeneous growth medium for further experiments. Diagrams to the left of the figures are representation of the experimental setup, each were coded with the color of the medium that supported the growth of each cell type.

|  | **Different types of growth mediums** | | | | | | **Different compositions of SFs, STFs, OBs growth mediums** | | | | | | | |
| --- | --- | --- | --- | --- | --- | --- | --- | --- | --- | --- | --- | --- | --- | --- |
|  | **SFs medium** | | **STFs medium** | | **OBs medium** | | **[2:1:1]** | | **[1:2:1]** | | **[1:1:2]** | | **[1:1:1]** | |
| **Condition**  **Cell type** | TCE | SCE | TCE | SCE | TCE | SCE | TCE | SCE | TCE | SCE | TCE | SCE | TCE | SCE |
| **SFs** | 0.91 ± 0.01 | ****1.52 ± 0.01 | 0.44 ± 0.03 | ****1.12 ± 0.02 | 1.62 ± 0.05 | ***1.94 ± 0.02 | 1.01 ± 0.17 | **1.61 ± 0.06 | 0.65 ± 0.1 | ***1.29 ± 0.05 | 1.42 ± 0.07 | **1.92 ± 0.07 | 0.99 ± 0.2 | *1.48 ± 0.06 |
| **STFs** | 1.04 ± 0.05 | ***1.69 ± 0.05 | 0.79 ± 0.24 | *1.33 ± 0.24 | 1.23 ± 0.11 | **1.71 ± 0.12 | 1.31 ± 0.12 | *1.61 ± 0.07 | 0.84 ± 0.28 | *1.48 ± 0.08 | 0.87 ± 0.17 | *1.22 ± 0.56 | 1.02 ± 0.12 | *1.5 ± 0.3 |
| **OBs** | *1.56 ± 0.21 | 1.12 ± 0.17 | *1.15 ± 0.04 | 0.64 ± 0.2 | *2.41 ± 0.51 | 1.44 ± 0.18 | *1.67 ± 0.31 | 0.97 ± 0.22 | *1.24 ± 0.31 | 0.61 ± 0.17 | **1.99 ± 0.2 | 1.31 ± 0.06 | *1.82 ± 0.17 | 1.2 ± 0.3 |

**Table S1: The growth of SFs, STFs, and OBs in respect to medium type in a TCE and SCE (Numerical representation of the graphical data).** The developed tri-culture system was utilized to determine a heterogeneous optimal growth medium that would sustain the growth of the three different cell types in a tri-culture environment. Data indicate that osteoblasts (OBs) medium provided an optimal growth medium for the three different cell types in a tri-culture environment. Regardless of the medium type, both SFs and STFs showed better growth in the SCE compared to the TCE. OBs’ growth was better in the TCE compared to the SCE. Numbers indicate the average increase in fold of three repeated experiments between days 3 and 7 (*n* = 3 per every experiment and *n* = 9 per fold number; Student t-test, *P < 0.05, **P < 0.01, ***P < 0.001, ****P < 0.0001 compared to the TCE or SCE for the same cell and medium types).


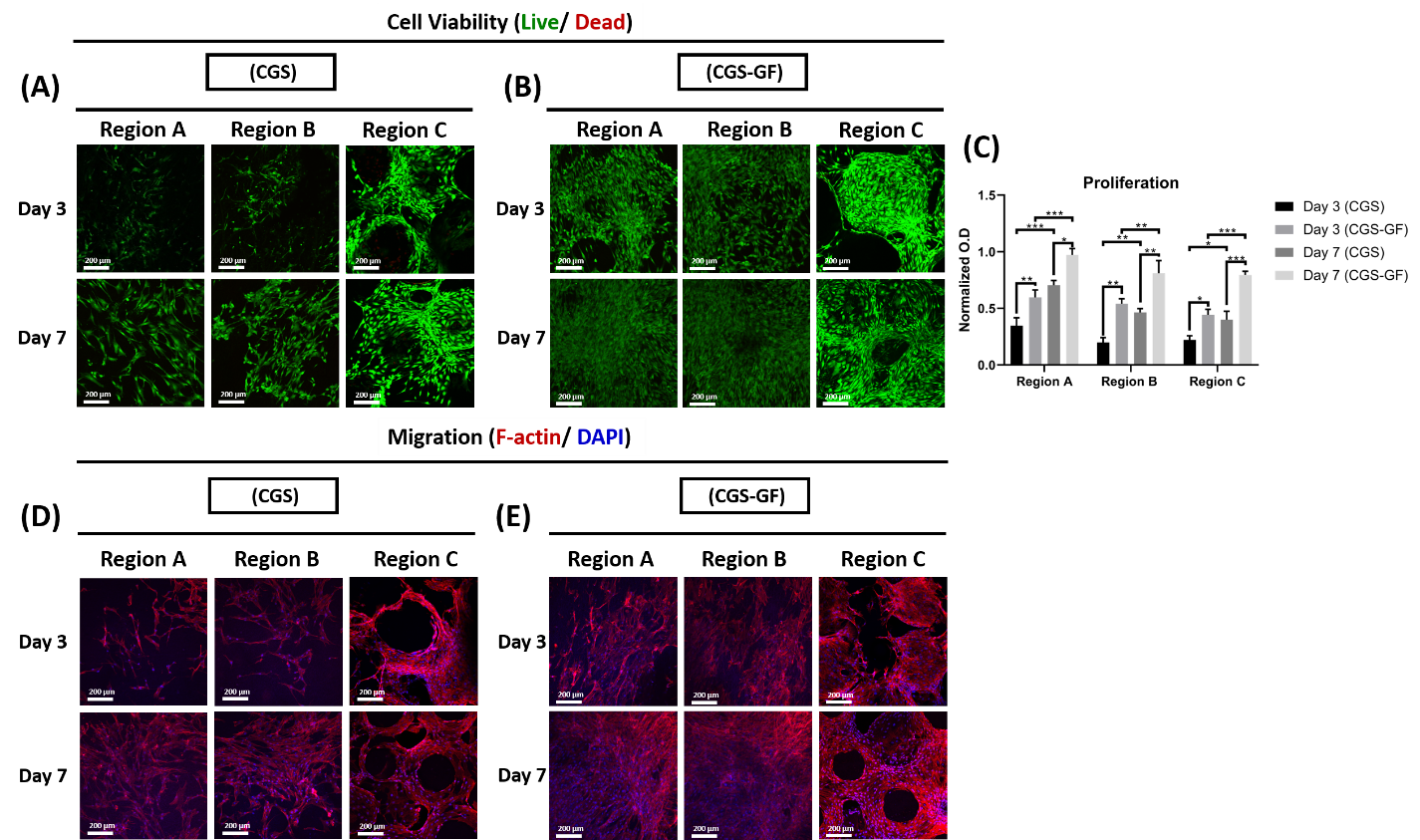


**Fig. S7: A direct comparison between CGS and CGS-GF highlighting the effects of growth factors addition on the region-spesific cell viability, proliferation and migration during the heterogeneous culture. (A)** Representative live/dead stained images for regions A, B and C in the CGS and **(B)** CGS-GF groups at 3 and 7 days (*n* = 3, and 4-6 random fields/ sample). **(C)** Proliferation of SFs, STFs, and OBs in regions A, B and C, respectively in the CGS and CGS-GF groups at 3 and 7 days (*n* = 3, ANOVA and *post hoc* Tukey test, *P < 0.05, **P < 0.01, ***P < 0.001). **(D)** Representative F-actin staining images for regions A, B and C in the CGS and **(E)** CGS-GF groups at 3 and 7 days (*n* = 3, and 4-6 random fields/ sample).


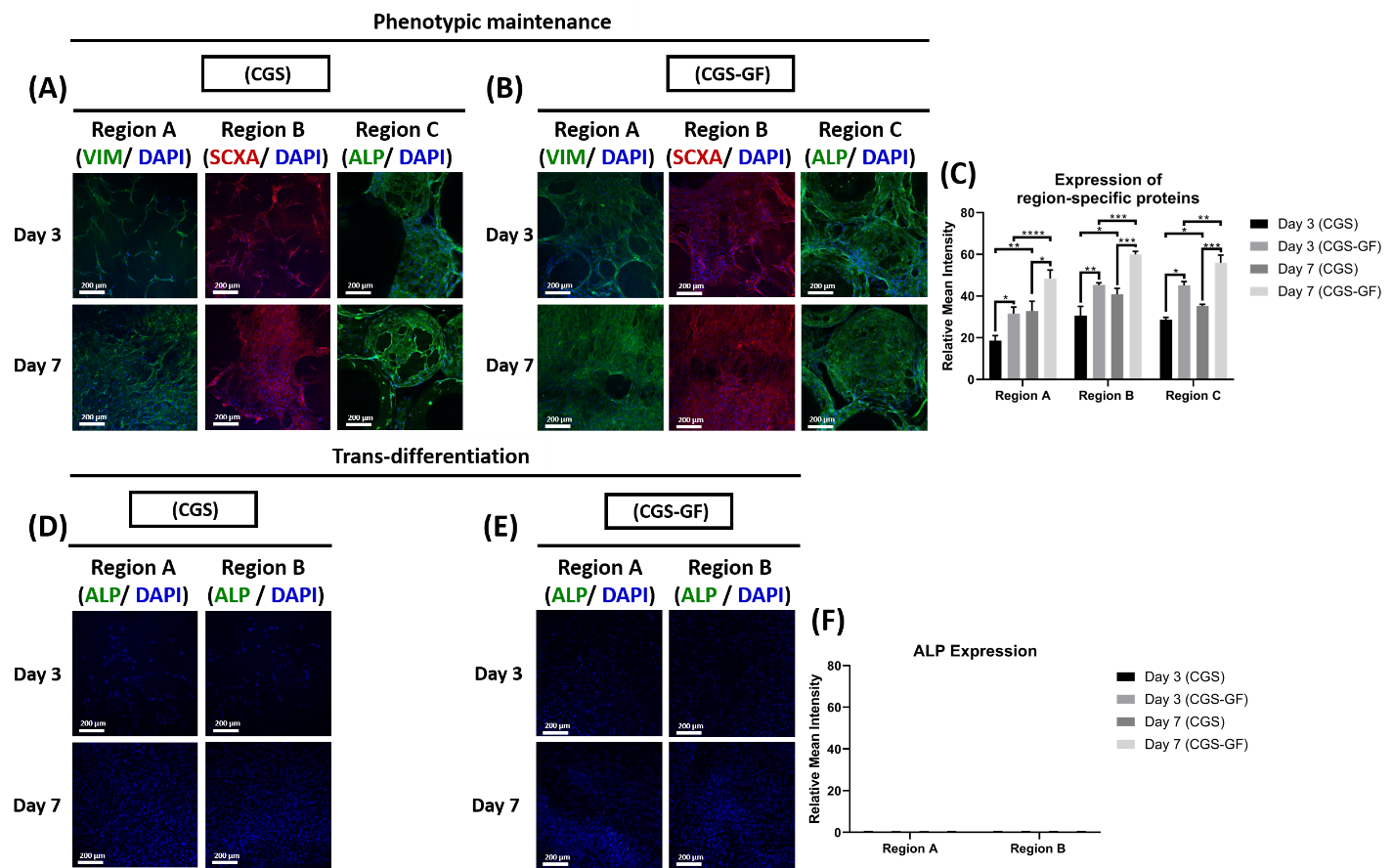


**Fig. S8:** **A direct comparison between CGS and CGS-GF highlighting the effects of growth factors addition on the region-spesfic protein expression levels and phenotypic maintaianace during the heterogeneous culture. (A)** Immunofluorescent staining of regions A, B, and C for VIM, SCXA, and ALP, respectively in the CGS and **(B)** CGS-GF groups at 3 and 7 days and **(C)** quantifications of VIM, SCXA and ALP expression from regions A, B and C in the CGS and CGS-GF groups at 3 and 7 days (*n* = 3, and 4-6 random images/ sample, ANOVA and *post hoc* Tukey test, *P < 0.05, **P < 0.01, ***P < 0.001, ****P < 0.0001). **(D)** Immunofluorescent staining of regions A and B for ALP in the CGS and **(E)** CGS-GF groups at 3 and 7 days and **(F)** quantifications of ALP expression from regions A and B in the CGS and CGS-GF groups at 3 and 7 days (*n* = 3, and 4-6 random images/ sample, ANOVA and *post hoc* Tukey test).
